# Optimization of cervical cord atrophy measurement using a real-world, multicentre dataset in multiple sclerosis

**DOI:** 10.3389/fneur.2025.1657484

**Published:** 2025-11-26

**Authors:** Carsten Lukas, Barbara Bellenberg, Ferran Prados, Paola Valsasina, Katrin Parmar, Iman Brouwer, Deborah Pareto, Alex Rovira, Jaume Sastre-Garriga, Claudia A. M. Gandini Wheeler-Kingshott, Michael Amann, Maria A. Rocca, Massimo Filippi, Marios C. Yiannakas, Eva M. M. Strijbis, Frederik Barkhof, Hugo Vrenken

**Affiliations:** 1 Institute of Neuroradiology, St. Josef Hospital, Ruhr-University Bochum, Bochum, Germany; 2 Department of Neurology, St. Josef Hospital, Ruhr-University Bochum, Bochum, Germany; 3 Department of Medical Physics and Biomedical Engineering, Centre for Medical Image Computing (CMIC), UCL, London, United Kingdom; 4 Department of Neuroinflammation, Faculty of Brain Sciences, UCL Queen Square Institute of Neurology, Queen Square MS Centre, University College London, London, United Kingdom; 5 e-Health Centre, Universitat Oberta de Catalunya, Barcelona, Spain; 6 Neuroimaging Research Unit, Division of Neuroscience, IRCCS San Raffaele Scientific Institute, Milan, Italy; 7 Department of Medicine, Neurological Clinic and Policlinic, University Hospital Basel, Basel, Switzerland; 8 Department of Radiology and Nuclear Medicine, Amsterdam UMC, Amsterdam, Netherlands; 9 Section of Neuroradiology, Department of Radiology, Hospital Universitari Vall d'Hebron, Barcelona, Spain; 10 Department of Neurology, Multiple Sclerosis Center of Catalonia (Cemcat), Hospital Universitari Vall d'Hebron, Barcelona, Spain; 11 Department of Brain & Behavioral Sciences, University of Pavia, Pavia, Italy; 12 Brain Connectivity Center, IRCCS Mondino Foundation, Pavia, Italy; 13 Medical Image Analysis Center (MIAC), Basel, Switzerland; 14 Neurology Unit, IRCCS San Raffaele Scientific Institute, Milan, Italy; 15 Vita Salute San Raffaele University, Milan, Italy; 16 Neurorehabilitation Unit, IRCCS San Raffaele Scientific Institute, Milano, Italy; 17 Neurophysiology Service, IRCCS San Raffaele Scientific Institute, Milano, Italy; 18 Department of Neurology, Amsterdam UMC, Amsterdam, Netherlands

**Keywords:** CSA, cross-sectional area, cervical cord, atrophy, multiple sclerosis, segmentation software, optimization MRI

## Abstract

**Background:**

Cervical cord atrophy is linked to disability in multiple sclerosis (MS). Cervical cord cross-sectional area (CSA) measurement for atrophy quantification using magnetic resonance imaging (MRI) has been technically validated, but information about effects of methodological choices on associations of CSA with clinical variables is lacking.

**Aim:**

Assessing how image acquisition, cord level selection, CSA normalization and segmentation software affect measurement variance, separation of clinical groups, correlations with clinical scores, and to formulate recommendations for future study designs.

**Methods:**

Head and neck 3D-T1-weighted MRI of people with MS (pwMS, *N* = 85) and healthy controls (HC, *N* = 19) from five European centers. CSA measurements encompassed four methods (Active surface method ASM, NeuroQLab, SCT-Propseg and SCT-Deepseg), at two different levels of the cervical cord: C1-2 and C1-7 and normalization using four methods, based on cervical dimensions. Coefficient of variation (CV) of CSA was assessed in HC. In MS, Spearman correlations of CSA with EDSS were assessed. Separation between relapsing (rMS) and progressive MS (pMS) was quantified by area-under-the-curve (AUC) from receiver-operator-characteristic analysis.

**Results:**

For all combinations of imaging, cord level, and segmentation software, unnormalized CSA differed between HC and pMS. CV in HC varied between 10.5 and 13.5% for unnormalized CSA and was lower for CSA normalized by C1-C2 (range: 9.4–12.0%) and C1-C3 vertebral height (8.6–12.6%). Unnormalized and normalized CSA correlated with EDSS scores for all measurement combinations (Spearman’s rho between −0.646 and −0.372, all corrected *p* < 0.001); correlations were stronger for CSA measured at vertebral level C1-7 than C1-2, and stronger for normalized than unnormalized CSA. Mean AUC for separating rMS from pMS ranged between 0.685 and 0.877, with higher AUC for CSA measured at the C1-7 than at the C1-2 vertebral level, and for normalized compared to unnormalized CSA.

**Conclusion:**

Clinical performance of CSA quantification regarding discrimination between rMS and pMS and correlations with EDSS was better for whole cervical cord (C1-7) than for C1-2 measurements, and for normalization by C1-C2 or C1-C3 vertebral height. Based on the quantitative results of this exploratory multi-center study and on previous literature, we formulated recommendations to support future study design decisions.

## 1 Introduction

Spinal cord (SC) atrophy is a common and clinically relevant aspect in multiple sclerosis (MS) reflecting axonal loss, gliosis or demyelination in both lesions and normal appearing tissue (1–3). On magnetic resonance imaging (MRI), people with MS (pwMS) exhibit SC atrophy compared to healthy controls (HC), which is more severe in progressive MS (pMS) than in relapsing–remitting MS (RRMS) (1, 3–7). Cervical SC atrophy has received most attention because the cervical portion of the cord is most readily accessible through MRI, and several image analysis software programs are available for its quantification (8–11). Cervical SC atrophy has previously been explored reliably in large MS cohorts using these software tools (4, 12–14). Cervical SC atrophy progresses faster than brain atrophy and correlates with future disability worsening (1, 12) and future conversion from RRMS to pMS (15, 16).

Although the clinical relevance of cervical SC atrophy is abundantly clear, and measurement of cervical cord cross-sectional area (CSA) is seen by some as a strong candidate to be part of future regular MS imaging (17), there are no clear evidence-based guidelines on how to implement CSA measurement. Previous validation studies have addressed many technical aspects, by quantitatively investigating how measurement of cervical cord CSA is affected by the choice of scanner (18, 19), the choice between head and cord imaging (18, 20), the choice of the cord level (18), the choice of image analysis software (18, 19, 21, 22), and the presence of MS lesions in the cervical cord (19); as well as quantifying the effect of gradient nonlinearity distortion (23), the magnitude of scan-rescan error (19), the smallest detectable change (18, 24) and the accuracy compared to manual segmentations (19). To account for anatomical inter-individual differences, several normalization methods have been proposed and investigated (25–28). While technical aspects have therefore been extensively addressed, the effect of methodological choices on the clinical validity of the resulting CSA measurements has not received much attention.

Therefore, to complement the technical validation studies, we performed a real-world multi-center study in pwMS and HC to investigate the clinical validity of CSA measurements. The real-world scenario implied that all centers used 3D T1w MRI sequences with 1 mm resolution, while the optimization and sequence timings were adjusted according to the individual practices of each facility. Specifically, we quantitatively investigated the effect of choices of head or neck imaging, cord level, image analysis software, and normalization method on the associations of CSA with clinical disability measures in MS, the separation between clinical groups, and the variability in HC.

## 2 Methods

### 2.1 Overview of the study

**Figure 1:**
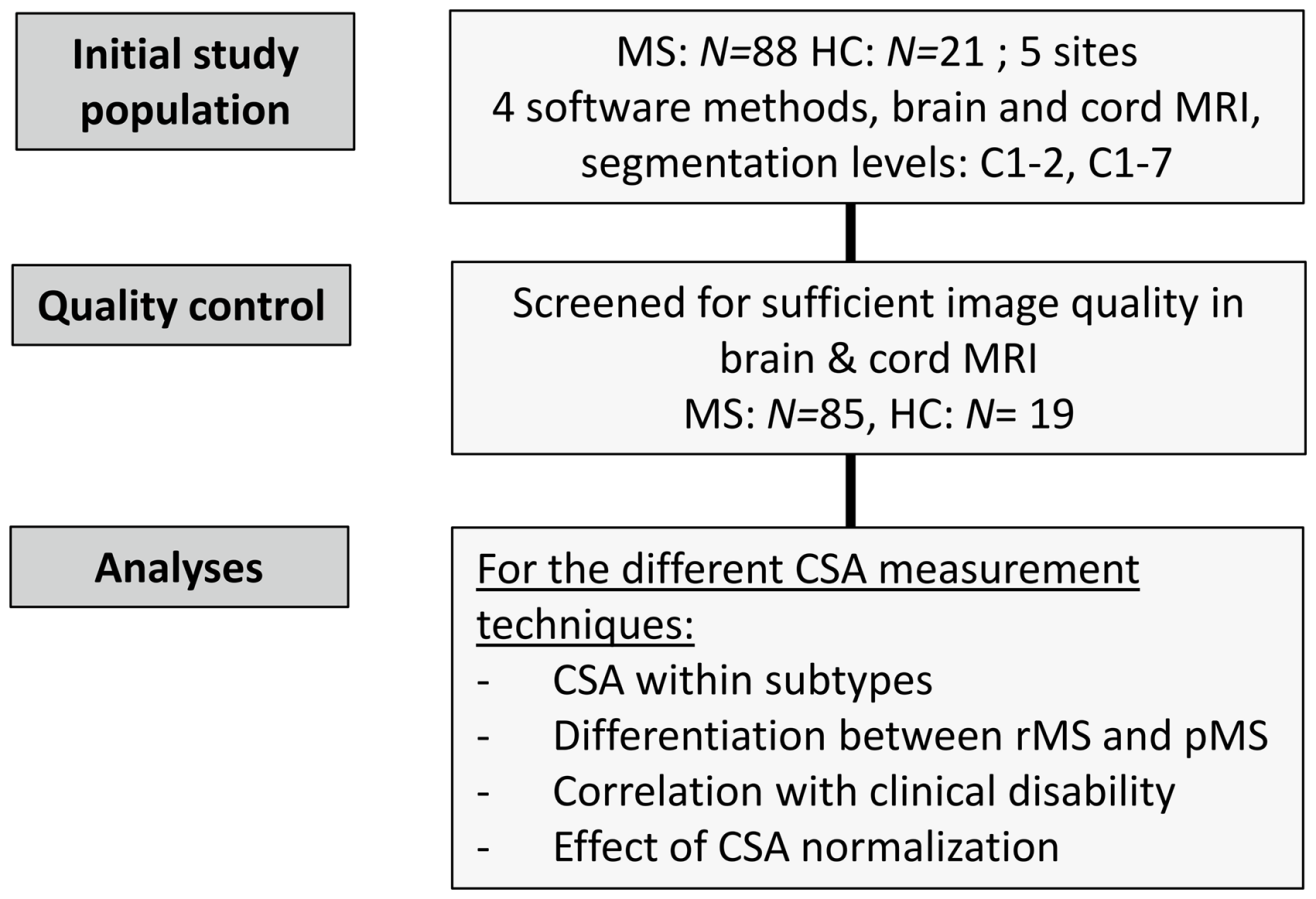
Flowchart showing the study processes and how the final sample sizes were obtained.

The flowchart in Figure 1 summarizes the different study processes leading to the final patients’ and HC samples. Patients and HC underwent MRI and a clinical examination (for patients only).

### 2.2 Participants

People with MS and HC were recruited at five European centers between 2010 and 2016: (1) = Amsterdam University Medical Center (UMC), The Netherlands; (2) CEM-Cat, Hospital Universitari Vall d’Hebron Barcelona, Spain; (3) St. Josef Hospital, Ruhr-University of Bochum, Germany; (4) Neuroimaging Research Unit, IRCCS San Raffaele Scientific Institute, Milan, Italy; (5) Queen Square Institute of Neurology, University College London (UCL), United Kingdom. Data from some of the participants in this study were also used in another MAGNIMS study (5), to minimize the burden on study participants.

#### 2.2.1 Ethics approval

This study was approved by the local Ethics Boards at each center; written informed consent was obtained from all subjects prior to study participation.

#### 2.2.2 Inclusion and exclusion criteria

Inclusion and exclusion criteria were the same as in the work by Rocca and colleagues (5): briefly, patients had either clinically isolated syndrome (CIS) suggestive of MS with MRI and clinical assessment within 3 months after their onset of symptoms, or definite MS of RRMS, primary progressive (PPMS) or secondary progressive (SPMS) phenotype under stable medication during the last 6 months and no corticosteroid application within 4 weeks before inclusion. Exclusion criteria for the HC group were neurological diseases, history of brain or spinal cord trauma, diabetes or cardiovascular diseases, drug abuse, or MRI contraindications.

### 2.3 Clinical examination

Disability status, according to the Expanded Disability Status Scale (EDSS) score (29) was assessed by an experienced neurologist.

### 2.4 MRI acquisition and analysis

Using combined neurovascular head and neck matrix coils, both brain imaging and cervical cord imaging were performed at 3 T, each including a sagittal, magnetization-prepared, high-contrast, 3D T1-weighted (3D-T1w) gradient-echo sequence, with isotropic spatial resolution of 1x1x1mm^3^. Care was taken to position the participants reproducibly straight with the midsagittal plane of brain and cervical cord parallel to the magnets z-axis. The brain 3D-T1w covered at least the C1-C3 vertebral levels, and the cord 3D-T1w covered the entire cervical SC. Acquisition was optimized in each center; Supplementary Table S1 provides acquisition details. The centers used MRI at 3 Tesla from three different vendors. The study design was intended to reflect a real-world multi-center scenario without the exact specification of the sequence design and timing, but with standardization for image geometry. The brain 3D-T1w sequences, but not necessarily the cord 3D-T1w sequences were part of the clinical protocols in all centers. No major scanner hardware or software upgrades occurred during the study. Non-linear gradient distortion effects were corrected using vendor-specific 3D geometric distortion correction procedures (23).

#### 2.4.1 Quality control

Patients and HC were included if brain and cervical SC imaging were both available. The MRI examinations and cord segmentations were checked and cases were excluded if insufficient image quality (excessive noise, image artefacts) was found.

#### 2.4.2 Image analysis methods

Four different image analysis software methods were used to quantify CSA: two semi-automatic methods, ASM [Active Surface Method named “cord finder” tool in JIM, v. 7.0, licensed by Xinapse Systems, Colchester, UK; http://www.xinapse.com, (4, 10)] and NeuroQLab [NQL, v4.01 Fraunhofer-Mevis, Bremen, Germany; license freely available for research purposes upon request from Fraunhofer-Mevis (3, 11)]; and two automated methods, SCT-PropSeg (30) and SCT-DeepSeg (9), both of which are freely available with the Spinal cord Toolbox (SCT, v. 5.6) [https://sourceforge.net/projects/spinalcordtoolbox/, (8)]. Details of the software and of the parameter settings used in this study can be found in the Supplementary material.

#### 2.4.3 Cord level selection for mean CSA estimation

**Figure 2:**
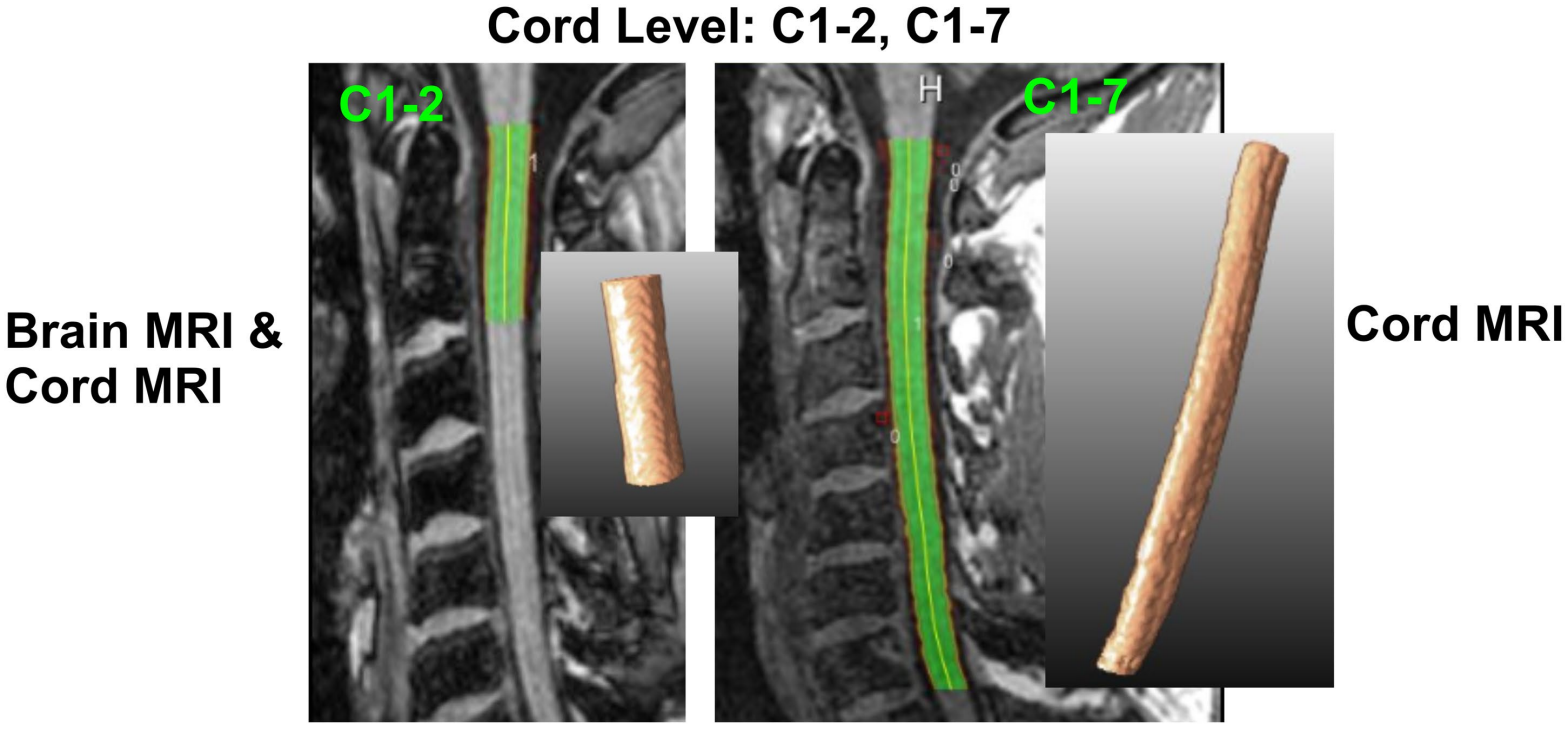
Definition of cervical cord level measurements on brain and cord MRI. C1-C2: upper cervical cord from top of C1 to bottom of C2 vertebra, C1-C7: entire cervical cord between top of C1 and bottom of C7 vertebral level. We show examples of 2D and 3D segmentations of the cervical cord based on the NQL method.

The C1-2 and C1-7 cord sections were chosen according to previous literature (3, 4, 22), and to match the included image analysis methods. On both head and spinal cord 3D-T1w images, CSA was measured for the full C1-2 range (upper cervical cord); on spinal cord 3D-T1w images alone, CSA was also measured for the full C1-7 range (whole cervical cord), as illustrated in Figure 2. For the semi-automated image analysis methods ASM and NQL, these sections were manually defined using anatomical references: the top of the dens for the upper boundary, and the endplate of the caudal vertebra (either C2 or C7) for the lower boundary (3, 4, 20). For the automated image analysis methods SCT-PropSeg (SCT_PS) and SCT-DeepSeg (SCT_DS), these sections were defined using SCT’s automated vertebral labeling of the cord [sct_label_vertebrae; (31)].

#### 2.4.4 CSA normalization

Four different CSA normalization measures were chosen based on the spine skeleton, as suggested in previous studies, to generate potential normalization variables from the spinal examination rather than relying on an additional brain analysis (25–27). We derived the normalization measures from a mid-sagittal view of the 3D-T1w cord MRI for each participant: (1) the height of the C1/C2 vertebra, measured from the tip of C1 to the lower border of the C2 vertebral body in the middle of its anterior–posterior extension; (2) the height of the C1-C3 vertebrae, measured as (1) but down to the lower border of C3 instead of C2; (3) the entire cervical cord length (C1-C7), extracted from the output of the ASM method; and (4) the sagittal area of the C3 vertebra, which was defined from the height of C3 at its anterior margin (height_a_), its height at its posterior margin (height_p_), and its anterior–posterior diameter (diameter_a–p_), as follows (Equation 1):


$$C3\;\text{vertebral area}=\frac{\left({\text{height}}_{a}+{\text{height}}_{p}\right)}{2}\cdot {\text{diameter}}_{a-p}\;$$ (1)

The vertebral heights and dimensions were measured using the ITK-SNAP software viewer (available at www.itksnap.org). For each normalization measure, we calculated the normalized CSA (nCSA) by dividing CSA by the subject-specific normalization measure, and multiplying by the average of that normalization measure in the healthy control group (Equation 2):


$${\text{nCSA}}_{\ldots }=\mathrm{CSA}\cdot \frac{\mathrm{HC}\;\text{average of normalization measure}}{\text{subject}\prime s\;\text{normalization measure}}$$ (2)

in (2) please insert a . for multiplication between CSA and the fraction lineinsert mid dot here to yield nCSA_C1-C2_, nCSA_C1-C3_, nCSA_C1-C7_, and nCSA_C3-area_, respectively.

### 2.5 Statistical analyses

Statistical analyses except for the DeLong’s analyses, ROC and covariate adjusted ROC analyses and linear mixed effects models, were performed using SPSS (IBM, SPSS V. 25). DeLong’s analyses, ROC and covariate-adjusted ROC (AROC), and linear mixed effects models were calculated using R (available at https://cran.r-project.org/web/packages/). Results were considered significant at *p* < 0.05. Unless specified otherwise, all statistical analyses were performed separately for each methodological combination, i.e., for each included combination of imaging setup (head or cord imaging), cord level (C1-2 or C1-7), image analysis software (ASM, NQL, SCT_PS or SCT_DS), and normalization measure (C1-C2 height, C1-C3 height, C1-C7 length, C3 vertebral area, or unnormalized).

We pooled the data of patients with CIS or RRMS into one relapsing MS (rMS) group, and the data of patients with SPMS or PPMS into a progressive MS group (pMS), to achieve sufficiently large sample sizes per subgroup. Normal distribution of the variables was evaluated using Shapiro–Wilk testing. Demographic variables were reported and compared as appropriate.

First, to provide insight in the data from different methodological combinations, mean and standard deviation of unnormalized CSA were calculated. To assess the separation between the groups (MS and HC, and rMS, pMS and HC) we calculated effect sizes using eta squared (η^2^) as effect size measure and interpreted η^2^ > 0.14/>0.06/>0.01 as strong/medium/small effects (32). The significance of differences in CSA between MS and HC, and between rMS, pMS and HC, was first investigated for each methodological combination using separate analyses of variance (ANOVA) adjusting for age and sex and correcting for multiple comparisons (between the three subgroups with Bonferroni correction, and with correction for false discovery rates between methodologies using Benjamini-Hochberg correction across methods, MRI type, vertebral level). In a second analysis, we included center as an additional confounder to account for possible influence of factors such as scanner type, acquisition protocol or demographics of the participants. We therefore investigated the group differences of CSA for each methodological combination using separate linear fixed effects models adjusting for age and sex with center as a random intercept, and also corrected for multiple comparisons between the three subgroups and methodological combinations using Bonferroni and Benjamini-Hochberg corrections.

To assess dependencies of CSA on age and, sex and center in the HC and in the MS group, we used a general linear model of CSA as dependent variable with inclusion of age as covariate, sex (male = 0, female = 1) as fixed factor and scanner (center no. 1,2,3,4,5) as a random factor. The same type of analysis was performed with EDSS as the dependent variable to investigate the interdependencies of EDSS with age, sex, and center.

Then, to assess agreement with previous reports, we compared CSA between the four different image analysis methods using a repeated measures ANOVA with the four software methods as within-subject factor and using Bonferroni correction for multiple comparisons. Furthermore, differences between the CSA measurements based on brain MRI C1-2 and cord MRI C1-2 were assessed using paired t-tests, while agreement between them was assessed by calculating intra-class correlation coefficients (ICC), separately for HC and MS.

Next, to investigate the performance of each different methodological combination for measuring CSA, we performed the following analyses: in the HC group, the association of each of the four CSA normalization measures with unnormalized CSA was assessed by Pearson’s correlation. Then, to assess the effect of the CSA normalization methods, the coefficient of variation (CV) in the HC group was calculated by dividing the group SD by the group mean; confidence intervals of CSA and SD were determined using bootstrapping with 1,000 resamples and the minimal to maximal ranges of CV were derived from them.

Each of the measures used for normalization of CSA was compared between groups (rMS, pMS and HC) using a separate analysis of variance, adjusting for age and sex, adjusting for multiple comparisons using Bonferroni correction between the subgroups.

In patients only, we assessed Spearman’s correlation between the EDSS score and CSA, for each methodological combination and normalization, and compared the resulting correlation coefficients.

Discrimination between rMS and pMS based on unnormalized and normalized CSA was assessed by area-under-the-curve (AUC) and AUC confidence intervals from receiver-operator-characteristic (ROC) analyses. In the primary analysis the *p*-values of significance of AUC differences between normalized CSA and unnormalized CSA were assessed pair-wise across the normalization strategies using the DeLong’s testing implemented in the pROC software package for R (available at https://cran.r-project.org/web/packages/pROC) and corrected for multiple comparisons between normalization strategies by Benjamini-Hochberg correction. In a secondary approach accounting for center effects, we included center as a covariate in covariate adjusted ROC analyses (AROC). An additional exemplary strategy-to-strategy contrast analysis comparing AUC of rMS and pMS differentiation while regarding normalization strategies x levels x MRI type x methods was performed for normalized and unnormalized CSA, NQL and ASM methods, and C1-2 and C1-7 levels and brain or cord MRI. Herein, all *p*-values derived from pairwise comparisons using DeLong’s testing were corrected for multiple comparison across all contrasts by application of Benjamini-Hochberg correction.

## 3 Results

### 3.1 Study population

Initially *N* = 88 pwMS and *N* = 21 HC, who had received a suitable MRI examination, were screened for recruitment. Three patients and two HC were excluded due to insufficient image quality, leaving *N* = 85 patients and *N* = 19 HC in the final cohort (Figure 1). Table 1 summarizes the demographic and clinical characteristics of the final dataset.

**Table 1:** Demography and clinical status of participants included in the analyses.

Site	*N*	HC/rMS/pMS [*N*]	Age/years	Sex % female	Age/years	Disease duration/years	Sex % female	EDSS
			HC		MS			
Cross-sectional data with brain and cord MRI								
1	25	7/18/0	54.8 ± 5.9	43	50.7 ± 7.7	19.7 ± 4.5	67	3.0 [1.5–6.0]
2	20	0/20/0	–	–	35.0 ± 9.4	0.2 ± 0.2	70	1.5 [0.0–4.0]
3	17	6/11/0	38.9 ± 10.2	50	37.9 ± 8.0	1.1 ± 0.9	55	2.0 [0.0–4.0]
4	26	6/16/4	33.1 ± 9.2	50	45.8 ± 10.4	15.2 ± 7.5	70	3.0 [1.0–7.0]
5	16	0/0/16	–	–	53.2 ± 10.0	19.0 ± 6.4	25	6.5 [3.0–7.0]
All sites	104	19/65/20	42.9 ± 12.5	47	44.4 ± 11.3	11.3 ± 10.6	59	3.0 [0.0–7.0]

N = number, HC healthy controls, rMS relapsing MS, pMS progressive MS; Age, disease duration: mean ± standard deviation; EDSS: median [minimum – maximum]; Site Numbers: 1 = Amsterdam UMC (The Netherlands); 2 = CEM-Cat Hospital Vall d’Hebron Barcelona (Spain); 3 = St. Josef Hospital; Bochum (Germany); 4 = San Raffaele Medical Insitute; Milan (Italy); 5 = London UCL (UK).

Patients with different MS phenotypes were not distributed evenly across the different centers (Table 1): centers 1, 2, 3 and 4 contributed patients with rMS, while pMS patients were recruited by center 5 and to a lesser extent by center 4. As a consequence, EDSS scores and disease durations also varied between centers; centers 1 and 4 included rMS patients with relatively long disease durations compared to center 2 and 3 (see also regression analyses of EDSS on age, sex and center in Supplementary Table S3). Because of this disbalance in the distribution of subtypes we did not primarily include center as a control variable in our analyses in the following sections, but rather investigated the effects of inclusion of center in additional secondary analyses.

### 3.2 Descriptive analyses

#### 3.2.1 CSA group comparisons and effect sizes between groups

**Figure 3:**
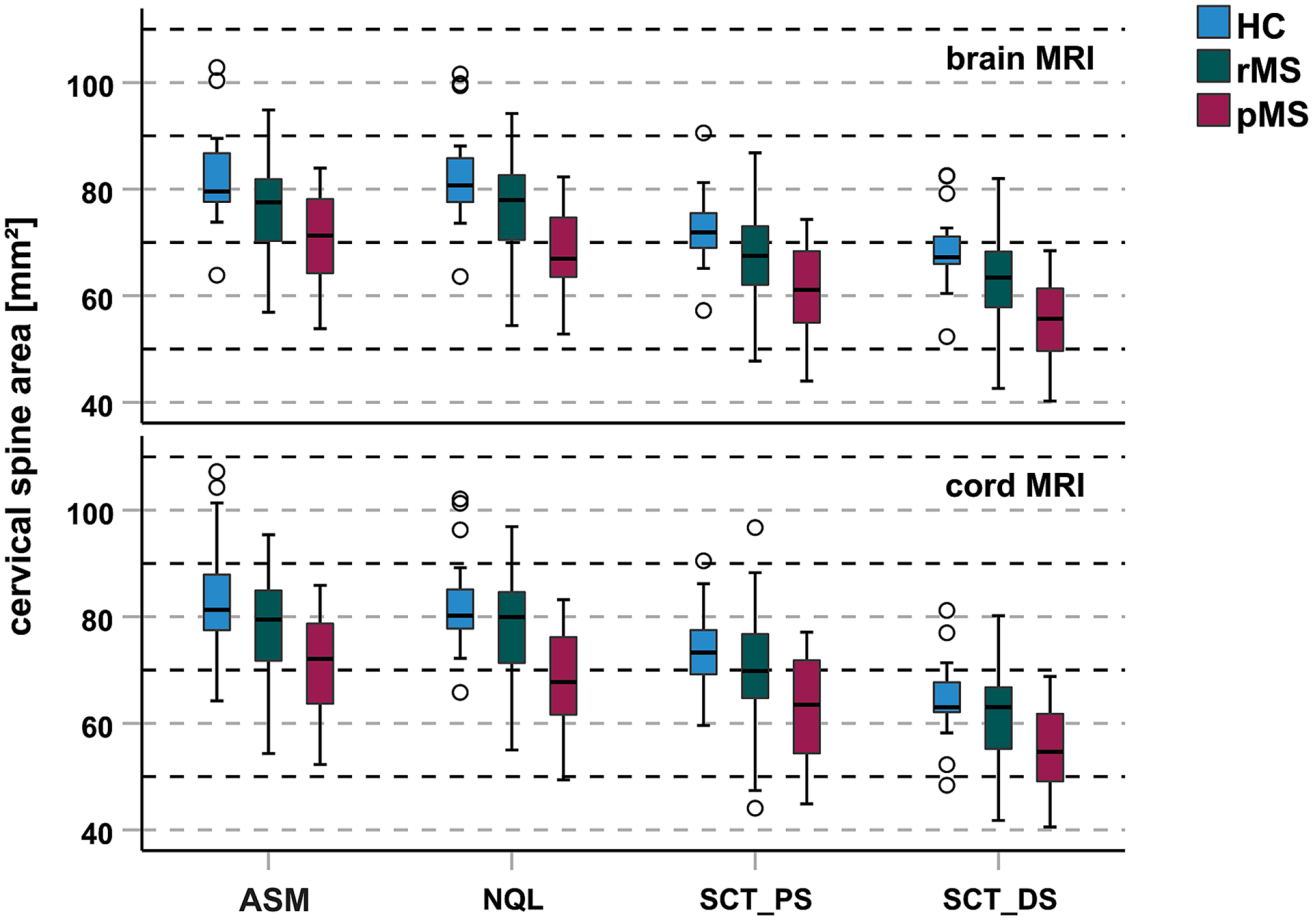
CSA determined at the C1-2 cervical cord level for the 4 different evaluation methods, without normalization, differentiated between the subgroups HC (blue), rMS (green) and pMS (red). Upper row: results extracted from brain MRI, lower row: results from spinal cord MRI; (boxes: median/interquartile range, error bars: minimum/maximum); Abbreviations: HC healthy controls, rMS relapsing MS, pMS progressive MS, SCT_DS SCT_deepseg, SCT_PS SCT_propseg, NQL NeuroQLab, ASM active surface method.

Mean values of unnormalized CSA showed a decline from HC to MS, and a stronger decrease in pMS compared to rMS in all methodological constellations (Table 2). As an example, Figure 3 shows unnormalized CSA determined at the C1-2 vertebral level for the four image analysis methods and for brain and cord MRI. The HC-MS differences were significant for CSA measured at C1-2 with brain MRI for all software methods. The effect sizes of HC-MS differences (representing a medium effect) were higher for brain MRI-derived C1-2 CSA than for C1-7 CSA for ASM, NQL, SCT_PS and SCT_DS. The mixed effects models controlling for age, sex and center related effects confirmed smaller CSA in MS than HC, while not significant (all corrected *p* > 0.05), but the effect sizes were still overall higher for brain and cord derived CSA at the C1-2 level than for CSA at the C1-7 level (Supplementary Table S2).

**Table 2:** Cord cross-sectional area (CSA unnormalized) in MS subgroups and HC for different software, cervical cord levels and brain or cord MRI.

Method/vertebral level	MRI type	CSA [mm^2^] mean ± standard deviation				HC - MS		HC – rMS - pMS			
		HC	MS	rMS	pMS	*p*	Effect size η^2^ (95% CI)	p (HC-pMS)^a^	p (HC-rMS)^a^	p (rMS-pMS)^a^	Effect size η^2^ (95% CI)
ASM											
C1-2	Brain	82.4 ± 9.6	75.4 ± 9.1	77.0 ± 8.5	70.2 ± 8.9	**0.024**	0.07 (0.01,1.00)	**<0.001**	0.473	**0.008**	0.15 (0.05, 1.00)
C1-2	Cervical cord	83.9 ± 11.1	76.6 ± 9.7	78.4 ± 9.1	70.9 ± 9.6	**0.024**	0.06 (0.01,1.00)	**<0.001**	0.473	**0.005**	0.15 (0.05, 1.00)
C1-7	Cervical cord	79.1 ± 9.9	72.6 ± 9.7	74.9 ± 8.5	65.3 ± 9.6	**0.034**	0.06 (0.01,1.00)	**<0.001**	0.644	**0.002**	0.17 (0.07, 1.00)
NQL											
C1-2	Brain	82.7 ± 9.6	75.0 ± 9.3	77.1 ± 8.7	68.2 ± 8.0	**0.024**	0.08 (0.02, 1.00)	**<0.001**	0.473	**0.001**	0.21 (0.10, 1.00)
C1-2	Cervical cord	82.4 ± 9.6	76.0 ± 10.0	78.4 ± 8.9	68.0 ± 9.3	0.060	0.04 (0.00, 1.00)	**<0.001**	0.999	**<0.001**	0.21 (0.09, 1.00)
C1-7	Cervical cord	82.7 ± 9.5	76.0 ± 10.7	79.1 ± 8.6	66.0 ± 10.6	0.060	0.04 (0.00, 1.00)	**<0.001**	0.999	**<0.001**	0.26 (0.14, 1.00)
SCT_PS											
C1-2	Brain	72.4 ± 7.0	66.3 ± 8.9	67.9 ± 8.5	61.3 ± 8.6	**0.024**	0.07 (0.01, 1.00)	**<0.001**	0.473	**0.002**	0.14 (0.04, 1.00)
C1-2	Cervical cord	74.0 ± 7.8	68.5 ± 10.3	70.5 ± 9.7	62.4 ± 9.8	0.060	0.04 (0.00, 1.00)	**0.003**	0.999	**0.017**	0.12 (0.03, 1.00)
C1-7	Cervical cord	74.8 ± 9.0	69.6 ± 11.9	72.7 ± 10.7	59.9 ± 10.8	0.109	0.03 (0.00, 1.00)	**0.001**	0.999	**0.002**	0.17 (0.06, 1.00)
SCT_DS											
C1-2	Brain	68.2 ± 7.6	61.5 ± 8.8	63.4 ± 8.3	55.8 ± 8.0	**0.024**	0.08 (0.01, 1.00)	**<0.001**	0.473	**0.005**	0.18 (0.07, 1.00)
C1-2	Cervical cord	64.5 ± 8.0	60.5 ± 9.1	62.3 ± 8.7	54.9 ± 8.1	0.152	0.02 (0.00, 1.00)	**0.003**	0.999	**0.005**	0.11 (0.02, 1.00)
C1-7	Cervical cord	62.9 ± 8.5	58.8 ± 8.2	60.7 ± 7.3	52.8 ± 8.1	0.109	0.03 (0.00, 1.00)	**<0.001**	0.999	**0.002**	0.15 (0.05, 1.00)

HC healthy controls, rMS relapsing MS, pMS progressive MS, SCT_DS SCT_deepseg, SCT_PS SCT_propseg, NQL NeuroQLab, ASM active surface method. *p*: *p*-values extracted by using univariate analyses of variance (ANOVA) adjusting for age and sex; a: pairwise comparisons with Bonferroni correction for multiple comparisons for MS subgroup analyses; all *p*-values were additionally corrected for multiple comparisons between methodologies (Benjamini-Holm correction across methods, MRI type, vertebral level); *p*-values < 0.05 are marked in bold font; Effect size η^2^: partial eta squared effect size of CSA differences between groups.

When comparing between HC, rMS and pMS (Table 2), CSA differed between HC and pMS, and between rMS and pMS, but not between HC and rMS, for all methodologies (software, MRI type, vertebral levels). Thus, the CSA differences observed between HC and MS were probably driven by the pMS group. Across the three subgroups the combined effect sizes, representing overall strong effects, were higher for NQL than for ASM, SCT_PS and SCT_DS, and higher for C1-7 than for C1-2 level when using ASM, NQL and SCT_PS (Table 2).

Inclusion of center as a confounder in the secondary analysis (Supplementary Table S2) considerably diminished the significances of the between group differences, with HC-rMS differences remaining significant (corrected *p* < 0.05) in NQL, mostly in ASM and in SCT_DS at brain MRI-derived C1-2 CSA, while the differences between rMS and pMS overall lost significance.

Our regression analysis of CSA on age, sex and scanner type showed that in the HC group there was no association of CSA with age, sex or scanner, while in the MS group CSA was negatively related with age and sex (smaller CSA at higher age and in female patients). Details are shown in Supplementary Table S2.

#### 3.2.2 Comparison between image analysis methods

Confirming published literature (18, 19, 22, 24), the different image analysis methods yielded systematically different CSA measurements, with highest CSA for NQL and ASM, lower CSA for SCT_PS and lowest CSA for SCT_DS (Figure 3; Table 2). Pairwise comparisons revealed significant differences of CSA between the different software methods, for both vertebral levels and in brain or cord MRI (all *p* < 0.001 after Bonferroni correction; details provided in Supplementary Table S3).

#### 3.2.3 Comparison between brain and cord MRI

Brain MRI yielded overall smaller CSA at the C1-2 level than cord MRI especially in the MS group with the ASM, NQL and SCT_PS analysis methods (all *p* < 0.05 in paired t-test), confirming our previous results (18), while average cord MRI CSA results were smaller than brain CSA with the SCT_DS method (Supplementary Table S4). Still, absolute agreement between CSA with brain and cord MRI was excellent for the ASM, NQL and SCT_DS methods in both MS and HC, and for SCT_PS only in the MS group, with ICC between 0.860 and 0.994 (33) (Supplementary Table S4).

#### 3.2.4 Normalization factors

The measured values of the four different normalization measures are provided in Supplementary Table S5. As reported in the supplement, the ANCOVA analyses showed no differences between the HC and both MS subgroups for the height of C1-C2 and height of C1-C3, but significant group differences, probably driven by a higher fraction of female participants in the rMS group, for height of C1-C7 and the C3 vertebral area.

For all four software methods, unnormalized CSA in the HC group was significantly correlated with the C1-C2 vertebral height, the C1-C3 vertebral height and with the length of the cervical cord (C1-C7), except for the ASM method. Correlations with the C3 vertebral area were not significant (Supplementary Table S6). In the HC group, CSA was not associated with age or sex (all *p* > 0.1); normalized CSA measures were also independent of age and sex in HC, except for nCSA_C3 area_, where weak associations with sex were observed in SCT_DS and SCT_PS (for all p: 0.05 > *p* > 0.015).

#### 3.2.5 Effects of normalization of CSA on variability in HC

CSA normalization by the C1-C2 vertebral height (nCSA_C1-C2_) led to the strongest reductions of the CV of CSA in HC compared to the unnormalized CSA (Table 3). On average, across all software and cord levels, normalization using the C1-C2 vertebral height decreased the CV by 1.3 percentage points, from 11.9 to 10.6%. In detail, CV reduction was seen for all cord levels in ASM, NQL and SCT_DS, and in SCT_PS for brain MRI at C1-2. Furthermore, in these measurement combinations, normalizing CSA by the C1-C3 vertebral height (nCSA_C1-C3_) resulted in smaller reductions of CV, while using the entire cervical cord length for normalization (nCSA_C1-C7_) generally did not clearly reduce CV. In contrast, in SCT_PS using cord MRI normalization with the C1-C3 vertebral height or the entire cervical cord length led to stronger CV reduction than C1-C2 vertebral height normalization. Conversely, normalizing by the C3 vertebral area increased CV in the HC group in all measurement combinations (Table 3).

**Table 3:** Coefficient of variation (CV) of unnormalized CSA and normalized CSA in the healthy control group using different normalization strategies (CV = standard deviation/mean).

Method/vertebral level	MRI type	CV in HC group [%] [min.–max. range]				
		CSA	nCSA_C1-C2_	nCSA_C1-C3_	nCSA_C1-C7_	nCSA_C3-area_
ASM						
C1-2	Brain	11.6 [5.1; 16.2]	10.9 [4.8; 15.4]	11.8 [4.7; 17.2]	12.6 [5.6; 18.6]	25.4 [8.4; 41.4]
C1-2	Cervical cord	13.2 [6.9; 17.9]	11.8 [6.1; 16.7]	12.6 [6.0; 18.2]	13.3 [6.4; 19.5]	24.5 [9.0; 40.0]
C1-7	Cervical cord	12.6 [6.9; 16.8]	11. [5.5; 16.2]	11.5 [5.9; 17.5]	12.2 [6.1; 18.6]	23.3 [9.3; 39.0]
NQL						
C1-2	Brain	11.7 [6.4; 15.9]	9.4 [5.0; 13.3]	10.3 [5.1; 14.9]	11.2 [5.9; 16.2]	22.9 [9.5; 36.8]
C1-2	Cervical cord	11.7 [6.1; 15.7]	9.4 [4.2; 13.9]	9.9 [4.4; 15.4]	10.8 [5.1; 16.8]	22. [7.8; 37.8]
C1-7	Cervical cord	11.5 [6.5; 15.4]	9.8 [4.6; 14.3]	10.1 [4.6; 15.2]	10.9 [5.2; 16.5]	22.2 [8.1; 36.8]
SCT_PS						
C1-2	Brain	9.7 [4.5; 14.2]	11.2 [6.0; 15.5]	11.2 [5.1; 16.7]	11.6 [5.; 17.9]	23.7 [8.0; 40.1]
C1-2	Cervical cord	10.5 [6.3; 13.8]	9.7 [6.1; 12.4]	8.6 [5.7; 10.8]	8.1 [5.5; 10.1]	17.1 [9.1; 24.1]
C1-7	Cervical cord	12. [7.3; 15.9]	11.8 [7.7; 15.3]	10.4 [7.2; 13.2]	9.7 [6.7; 12.3]	17.5 [10.3; 24.1]
SCT_DS						
C1-2	Brain	11.2 [6.3; 15.4]	9.4 [5.1; 13.1]	10.1 [5.3; 14.5]	11.2 [6.2; 16.3]	22.1 [8.9; 35.2]
C1-2	Cervical cord	12.4 [6.7; 17.2]	11.2 [6.8; 14.7]	11.9 [7.1; 16.0]	12.5 [7.2; 17.2]	23.1 [9.9; 36.6]
C1-7	Cervical cord	13.5 [7.4; 18.7]	12.0 [7.0; 15.3]	12.6 [7.0; 16.4]	12.8 [7.; 16.8]	23.8 [9.9; 36.5]

SCT_DS SCT_deepseg, SCT_PS SCT_propseg, NQL NeuroQLab, ASM active surface method; CV coefficient of variation = standard deviation of CSA/mean of CSA in [%]; min.-max. range minimum to maximum range of CV.

#### 3.2.6 Detecting correlations with EDSS in MS

**Figure 4:**
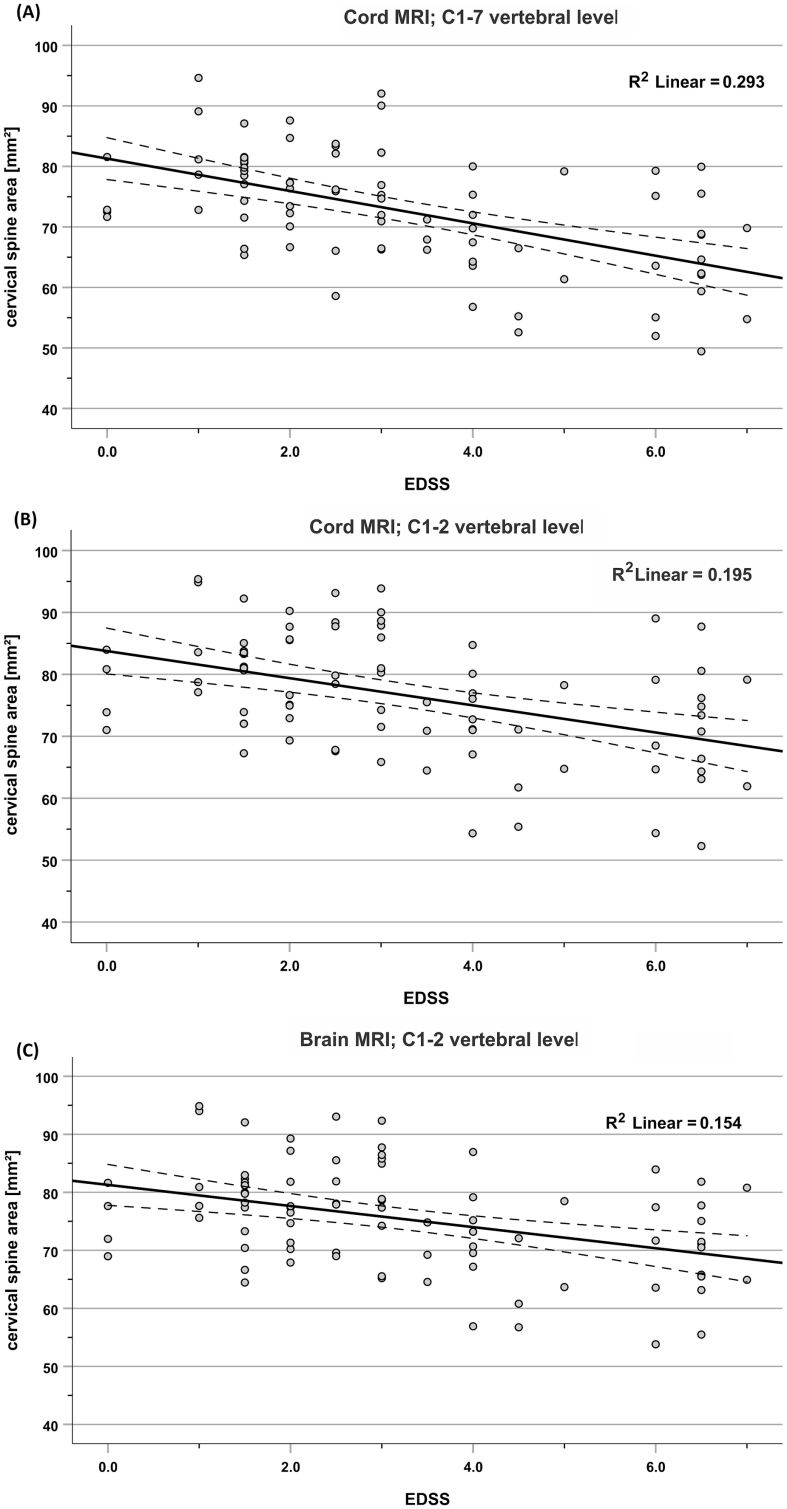
Association between unnormalized CSA and EDSS, shown exemplarily for the ASM method in cord MRI at C1-7 **(A)** and C1-2 **(B)** and in brain MRI at C1-2 **(C)**; solid lines: linear regression line; dotted lines: variability of the mean.

All measurement combinations showed significant correlations (all *p* < 0.001, corrected for multiple comparisons) of EDSS with normalized and normalized CSA. Correlation coefficients ranged between −0.372 and −0.603 for unnormalized CSA (Table 4). Figure 4 shows example scatterplots of EDSS and unnormalized CSA for the ASM method. These correlations were stronger for CSA determined from the entire cervical cord (C1-7) than CSA determined in the upper cervical cord (C1-2, brain and cord MRI).

**Table 4:** Spearman correlation coefficients and 95% confidence intervals of correlations of EDSS with CSA and normalized CSA using different normalization measures.

Method/vertebral level	MRI type	Normalization method				
		None	C1-C2 height	C1-C3 height	C1-C7 length	C3 area
ASM						
C1-2	Brain	−0.372 [−0.548, −0.165]	−0.515 [−0.66, −0.332]	−0.529 [−0.671, −0.349]	−0.476 [−0.632, −0.285]	−0.431 [−0.595, −0.233]
C1-2	Cervical cord	−0.438 [−0.6, −0.24]	−0.547 [−0.685, −0.371]	−0.535 [−0.676, −0.356]	−0.509 [−0.656, −0.325]	−0.441 [−0.603, −0.244]
C1-7	Cervical cord	−0.547 [−0.685, −0.372]	−0.616 [−0.737, −0.458]	−0.602 [−0.726, −0.44]	−0.568 [−0.701, −0.397]	−0.479 [−0.633, −0.289]
NQL						
C1-2	Brain	−0.444 [−0.605, −0.248]	−0.582 [−0.711, −0.415]	−0.589 [−0.716, −0.424]	−0.549 [−0.687, −0.372]	−0.463 [−0.62, −0.27]
C1-2	Cervical cord	−0.507 [−0.654, −0.322]	−0.609 [−0.731, −0.449]	−0.611 [−0.733, −0.452]	−0.587 [−0.715, −0.421]	−0.481 [−0.634, −0.291]
C1-7	Cervical cord	−0.543 [−0.682, −0.367]	−0.642 [−0.756, −0.491]	−0.617 [−0.737, −0.459]	−0.573 [−0.704, −0.403]	−0.484 [−0.637, −0.295]
SCT_PS						
C1-2	Brain	−0.376 [−0.554, −0.165]	−0.502 [−0.653, −0.313]	−0.501 [−0.652, −0.311]	−0.447 [−0.611, −0.246]	−0.436 [−0.602, −0.235]
C1-2	Cervical cord	−0.474 [−0.631, −0.281]	−0.58 [−0.711, −0.409]	−0.566 [−0.701, −0.393]	−0.538 [−0.68, −0.356]	−0.466 [−0.624, −0.271]
C1-7	Cervical cord	−0.603 [−0.728, −0.439]	−0.646 [−0.76, −0.494]	−0.638 [−0.754, −0.484]	−0.635 [−0.752, −0.478]	−0.552 [−0.69, −0.375]
SCT_DS						
C1-2	Brain	−0.508 [−0.657, −0.322]	−0.587 [−0.716, −0.418]	−0.591 [−0.719, −0.423]	−0.555 [−0.692, −0.379]	−0.498 [−0.649, −0.309]
C1-2	Cervical cord	−0.451 [−0.611, −0.254]	−0.573 [−0.705, −0.403]	−0.579 [−0.71, −0.41]	−0.536 [−0.677, −0.356]	−0.486 [−0.639, −0.296]
C1-7	Cervical cord	−0.479 [−0.634, −0.287]	−0.589 [−0.718, −0.421]	−0.573 [−0.706, −0.4]	−0.559 [−0.696, −0.384]	−0.472 [−0.629, −0.278]

All correlations were significant at *p* < 0.001 using Bonferroni-Holm correction for multiple comparisons (regarding segmentation methods, levels and normalization methods). SCT_DS SCT_deepseg, SCT_PS SCT_propseg, NQL NeuroQLab, ASM active surface method.

Normalization of CSA led to correlation coefficients of greater magnitude (Spearman’s rho between −0.431 and −0.646, Table 4) compared to unnormalized CSA (rho between −0.372 and −0.547), except for normalization using the C3 vertebral area (nCSA_C3-area_), with the strongest effect in nCSA_C1-C2_ and nCSA_C1-C3_. On average, across all software and cord levels, normalization using the C1-C2 vertebral height increased the magnitude of the correlation coefficients by 21.7% (average correlation coefficient changed from −0.478 to −0.582).

#### 3.2.7 Differentiation between rMS and pMS using ROC analyses

Results of the ROC analyses for the differentiation between rMS and pMS are provided in Table 5 and in Supplementary Figure S1, Supplementary Table S8. Across all measurement combinations, AUC values ranged between 0.685 and 0.877, representing moderate discriminating ability (34). We observed increases of the AUC with normalization, strongest when using the C1-C2 and C1-C3 vertebral heights. Higher AUC values were found for entire cervical cord (C1-7) than in upper cervical cord (C1-2) measurements in all methodologies, and overall highest AUC for NQL across the segmentation methods. These differences between AUC, assessed using pairwise DeLong testing between normalized and unnormalized CSA measurements, were not significant (corrected *p*-values > 0.05; Table 5). Still, AUC confidence intervals were all shifted up when normalizing CSA by the C1-C2 and C1-C3 vertebral heights in all segmentation methods.

**Table 5:** Receiver operating characteristic (ROC) analyses to determine the quality of differentiating between rMS and pMS subtypes for CSA and normalized CSA at baseline.

Method/vertebral level	MRI type	CSA	nCSA_C1-C2_		nCSA_C1-C3_		nCSA_C1-C7_		nCSA_C3-area_	
		AUC CI (95%)	AUC CI (95%)	*p*	AUC CI (95%)	*p*	AUC CI (95%)	*p*	AUC CI (95%)	*p*
ASM										
C1-2	Brain	0.692 [0.559, 0.826]	0.769 [0.645, 0.893]	0.248	0.77 [0.648, 0.893]	0.248	0.739 [0.620, 0.858]	0.367	0.774 [0.664, 0.884]	0.367
C1-2	Cervical cord	0.716 [0.586, 0.846]	0.777 [0.651, 0.902]	0.439	0.769 [0.645, 0.892]	0.439	0.752 [0.633, 0.872]	0.439	0.774 [0.666, 0.882]	0.429
C1-7	Cervical cord	0.77 [0.648, 0.891]	0.814 [0.706, 0.922]	0.667	0.807 [0.701, 0.913]	0.667	0.788 [0.685, 0.892]	0.667	0.799 [0.700, 0.899]	0.667
NQL										
C1-2	Brain	0.771 [0.655, 0.888]	0.84 [0.738, 0.942]	0.242	0.837 [0.737, 0.936]	0.242	0.812 [0.716, 0.908]	0.467	0.812 [0.716, 0.907]	0.554
C1-2	Cervical cord	0.791 [0.681, 0.901]	0.852 [0.751, 0.952]	0.488	0.843 [0.744, 0.942]	0.488	0.829 [0.738, 0.920]	0.507	0.825 [0.732, 0.918]	0.594
C1-7	Cervical cord	0.832 [0.737, 0.926]	0.877 [0.786, 0.968]	0.802	0.866 [0.775, 0.956]	0.802	0.834 [0.741, 0.926]	0.961	0.834 [0.741, 0.927]	0.961
SCT_PS										
C1-2	Brain	0.685 [0.552, 0.818]	0.779 [0.659, 0.898]	0.212	0.761 [0.640, 0.882]	0.224	0.728 [0.605, 0.850]	0.367	0.762 [0.651, 0.874]	0.367
C1-2	Cervical cord	0.711 [0.579, 0.843]	0.794 [0.678, 0.909]	0.280	0.777 [0.658, 0.897]	0.280	0.751 [0.632, 0.870]	0.339	0.784 [0.669, 0.898]	0.309
C1-7	Cervical cord	0.79 [0.678, 0.903]	0.84 [0.737, 0.942]	0.458	0.825 [0.722, 0.928]	0.458	0.822 [0.723, 0.921]	0.458	0.828 [0.731, 0.926]	0.458
SCT_DS										
C1-2	Brain	0.739 [0.615, 0.863]	0.806 [0.693, 0.918]	0.334	0.796 [0.687, 0.905]	0.334	0.763 [0.656, 0.871]	0.581	0.79 [0.688, 0.893]	0.581
C1-2	Cervical cord	0.722 [0.594, 0.850]	0.808 [0.700, 0.916]	0.068	0.805 [0.699, 0.910]	0.068	0.776 [0.673, 0.879]	0.251	0.795 [0.698, 0.893]	0.279
C1-7	Cervical cord	0.757 [0.628, 0.886]	0.823 [0.718, 0.929]	0.373	0.81 [0.702, 0.917]	0.373	0.803 [0.704, 0.902]	0.373	0.794 [0.695, 0.892]	0.599

We show the area under the curve (AUC), 95% confidence interval (CI) and corrected *p*-values of pairwise differences between AUC (DeLong tests) comparing normalized CSA with unnormalized CSA. Correction of *p*-values for multiple comparisons across normalization methods (Bonferroni) and methodologies (Benjamini-Hochberg). SCT_DS SCT_deepseg, SCT_PS SCT_propseg, NQL NeuroQLab, ASM active surface method.

An exemplary strategy-to-strategy contrast analysis of rMS and pMS differentiation accounting for normalization x MRI x levels x segmentation methods is shown for normalized and unnormalized CSA, brain or cord MRI, C1-2 and C1-7 levels and NQL and ASM methods in Supplementary Table S8. Therein, comparing normalized to unnormalized CSA, the corrected p-values of the DeLong testing confirmed non-significant AUC differences, at higher AUC for normalized CSA. Additionally, a tendency for higher AUC of rMS and pMS differentiation when using NQL compared to ASM for CSA and nCSA_C1-C2_ determination was confirmed (all *p* < 0.1), but neither the differences between measurement at the C1-2 or C1-7 vertebral level, nor between cord or brain MRI proofed significant in NQL or ASM for CSA or normalized CSA.

When including center as a confounder in the additional covariate adjusted ROC analyses, we received overall very low AUC (all AUC < 0.5, data not shown), so differentiation between rMS and pMS was not possible.

## 4 Discussion

This study systematically evaluated clinical validity of CSA measurements in a real-world, multi-center setting, providing actionable data and resulting recommendations to optimize measurements of the upper cervical cord CSA in MS. Specifically, the study quantitatively investigated the effect of choices between head or neck imaging, cord level, segmentation software, and normalization method, on the associations of CSA with clinical measures in MS, the separation between clinical groups, and the variability in healthy controls. These results add to previous literature on technical aspects of CSA measurement and taking into consideration all those results, this paper provides a set of recommendations for CSA measurement in various settings.

### 4.1 Importance of clinical validity and suitability of outcomes

With good accuracy and reproducibility (19) of CSA measurement techniques, CSA is a potentially potent marker of disease in MS: as evidenced by a meta-analysis performed by Casserly and colleagues, CSA has consistently been found to be decreased in MS compared to HC, (1), more so in pMS than rMS (1). Moreover, as evidenced by another meta-analysis performed by Song and colleagues, decreased CSA has also consistently been found to be moderately but significantly related to the severity of clinical disability as measured by the EDSS score (35). Therefore, optimal CSA measurements should ideally also yield maximum separation between clinical groups and the strongest correlation with EDSS scores. We have chosen this indirect validation to complement previous technical validations. However, confounding factors that might influence the relation between CSA measurements and EDSS, or the CSA differences between relapsing and progressive MS subtypes cannot be ruled out. Still, on the condition that the measurements are technically sound, optimized and stable, we regard these two relations as suitable for quantitatively comparing the clinical validity of different methodological approaches to measuring CSA.

### 4.2 Actionable recommendations on four key points

**Figure 5:**
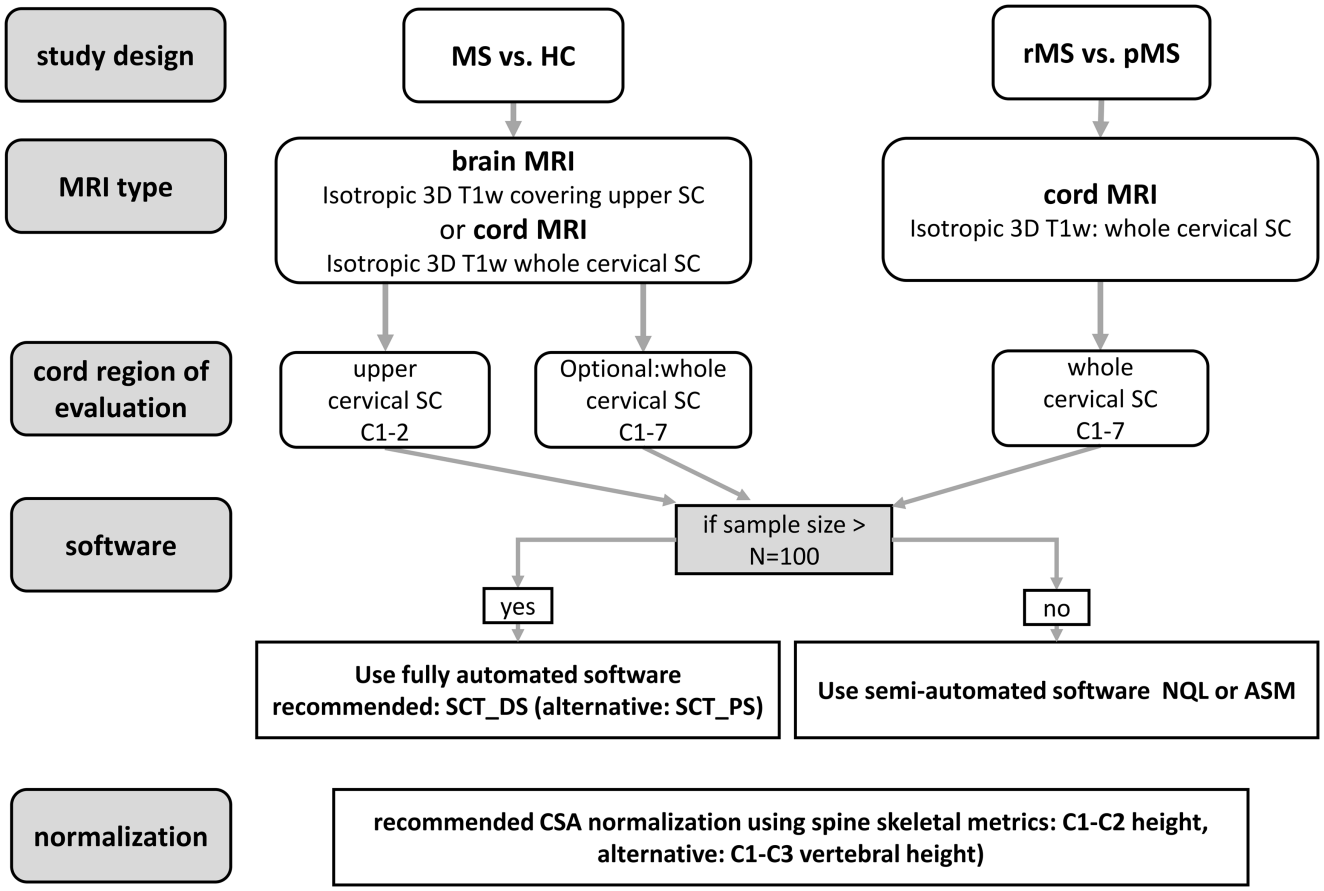
Recommendations for CSA quantification in the cervical cord of patients with MS.

This study provides actionable data on four important methodological points in CSA measurement: normalization, head or neck acquisition, cord level, and segmentation software. The recommendations derived from these results, from previous literature and from practical considerations, are summarized in the flowchart in Figure 5. Briefly, CSA should be normalized, and among spine-based metrics, C1-C2 height is preferable; if the studied cohort includes pMS, whole cervical cord imaging and C1-7 CSA measurement are preferred; and semi-automated segmentation software is preferable if practically feasible. The reasoning behind these recommendations is summarized in Supplementary Table S9. As regards cervical cord area, these recommendations complement the generic acquisition protocol that was recently proposed for cord image acquisition (36), thus further contributing to optimized CSA measurement, which is beneficial not only in MS but also in other diseases (1, 37, 38).

### 4.3 Normalization of CSA

Normalization of CSA values is used to overcome the high inter-subject variability of CSA present even in the absence of disease, which is partly anatomical in origin and may be partly related to differences in tilting of the spine in the MRI and to its positioning relative to the isocenter. This variability generally limits the sensitivity and specificity of detecting disease-related changes in the spinal cord in patients (26, 39). The present study found that CSA variability in HC was reduced when normalizing CSA by the heights of C1-C2 (on average by 11.1%) and C1-C3 and by the total cord length from C1 to C7 for most methodological combinations. Martin and colleagues similarly observed reduced variability in HC after normalizing by cervical cord length (27). Moreover, those effects were similar in size to those observed in the present study: their CV was reduced from 12.2 to 10.2% (27), and our results showed similar reductions, e.g., NQL C1-7 CV was 11.5% for unnormalized CSA and 9.8% for CSA normalized by C1-C2 vertebral height (Table 3). This enhances confidence in the use of vertebral heights as CSA-normalizing measures. Importantly, the current study adds the dimension of different cord segmentation methods, providing data allowing investigators to make their own informed decisions on which normalization method would be best, depending on the cord segmentation method to be used.

Conversely, normalizing by the C3 vertebral area resulted in a marked increase of CSA variability in all cases in the present study. That appears to be in contrast to findings of Papinutto and colleagues, who observed a reduction of variability by normalizing CSA by the combination of C3 vertebral area and intracranial volume (28). However, since the effect of C3 vertebral area was not assessed separately in that study, this apparent discrepancy is difficult to interpret. In general, the C3 vertebral area is also the most complex of the normalization measures used in this study introducing a higher variability (Supplementary Table S6), as it might depend on the image orientation, the tilting of the spinal cord and the method used to determine its dimensions, which might contribute to larger relative variability for C3 vertebral area than the other normalization measures.

Next, our results quantitatively indicated improved differentiation between the MS subgroups for all investigated normalization metrics and stronger correlations with disability (EDSS score) for all investigated normalization metrics except the C3 vertebral area. Especially the C1-C2 vertebral height and to a slightly lesser degree the C1-C3 vertebral height yielded consistent improvements (average increase of correlations with EDSS of 16.1% and average increase of AUC for discerning between rMS and pMS by 8.6% when using the C1-C2 vertebral height for normalizing CSA). Although the pairwise differences between the ROC curves of normalized and non-normalized CSA were non-significant when correcting for multiple comparisons (Table 5), a general trend towards improvement of AUC through normalisation by C1-C2 or C1-C3 vertebral height was evident in all segmentation methods and vertebral levels.

The effect of normalizing by C3 area is partly surprising: while (1) variability in healthy controls of C3 area-normalized CSA was increased, i.e., worse than that of unnormalized CSA, and (2) correlations with EDSS scores were either worsened or unchanged, by contrast (3) discrimination between rMS and pMS was notably better than for unnormalized CSA, with higher AUC (Table 5). While the improved group discrimination could be really due to CSA, confounding effects from the different distributions of sex and age in the rMS and pMS groups cannot be excluded. Height differences and age-related vertebral degeneration might explain part of the group discrimination observed based on C3 area-normalized CSA rather than MS-related spinal cord atrophy.

This work investigated a selection of measures for normalizing CSA values, but others such as spinal canal area (40) also exist and should be studied in future work. Furthermore, we selected spine-based normalization measures, to avoid having to rely on additional brain imaging and analysis, which would introduce additional variability, especially between sites. Nevertheless, there is some evidence that if appropriate brain MRI and analysis pipelines are available, CSA could also be normalized by other measures. Intracranial volume (ICV) is an important candidate normalization factor for CSA, which has been used by several larger studies, e.g., (16). ICV was found by Papinutto and colleagues to reduce CSA variability in healthy subjects in combination with C3 sagittal vertebral area (28). Similarly, the volumetric scaling factor calculated by FSL-SIENAX to normalize for head size, on its own was found to decrease CSA variability in healthy subjects (41). Conversely, Healy and colleagues found that adding ICV to their model normalizing spinal cord volume already correcting for cord length, yielded increased rather than decreased variability in HC (26). Bédard and colleagues considered volumes of brain structures (42) and saw the largest reduction of variability in HC for the combination of sex, thalamus volume, brain volume and the interaction between brain volume and sex. However, they explicitly cautioned against using volumes of brain structures to normalize CSA in diseases in which those brain structures are prone to atrophy (42).

### 4.4 Cord or brain imaging and cord level for image acquisition and analysis

The present study provides two novel insights regarding head or cord image acquisition and the cord level at which CSA is measured. First, that brain MRI-derived C1-2 CSA showed only slightly weaker associations with EDSS scores than CSA estimated using cord MRI. Strong associations between CSA and EDSS were seen with all segmentation methods, at the C1-2 and C1-7 vertebral levels, and for spinal cord and brain MRI acquisitions (for C1-2). The strongest relationships with EDSS scores were observed for CSA measured in the entire cervical cord (C1-7) rather than C1-2. We could speculate that the inclusion of a relatively large number of progressive patients might explain the stronger correlation with EDSS scores observed for CSA measurements from C1-7 than for those from C1-2. Higher EDSS (e.g., >3.5) is particularly influenced by the patient’s motor abilities (43) and motor impairments in the upper and lower extremities are associated with atrophy of the spinal cord. Some studies have found more pronounced atrophy in the caudal cervical cord in people with pMS (5, 7, 44), or faster progression of atrophy in caudal cervical cord (45), although others found no preferential atrophy in the caudal cervical cord but found atrophy to be more pronounced in PMS across the entire cervical cord (46). Caudal cervical cord atrophy has also been proposed as a measure predicting subsequent conversion from relapsing–remitting MS to secondary progressive MS (47).

Second, discriminating pMS from rMS based on C1-2 CSA worked similarly well using brain and cord MRI acquisitions (ROC analyses in Table 5 and Supplementary Table S8). Nonetheless, AUC values for discriminating rMS from pMS were typically slightly higher if C1-7 CSA was used, reflecting the higher prevalence of atrophy in the lower segments of the cervical cord in progressive MS compared to RRMS (5).

Additionally, in a large group of subjects from a large number of centers, the present study confirmed previous findings (18, 20, 48) that if gradient nonlinearity distortions are corrected (23), upper cervical CSA can be reliably quantified from brain MRI (48), as C1-2 CSA measured in brain and cord MRI showed good agreement by intra-class correlation. Still, CSA estimation using brain and cord MRI acquisitions should not be mixed as brain MRI results were slightly smaller than cord MRI results at the C1-2 level (Supplementary Table S5).

Despite using the vendors distortion correction this effect may be related to residual coil-edge effects that may particularly influence the CSA quantification based on brain MRI, since the upper cervical cord is located off-center in the sagittal images, at the periphery of the field of view (18).

### 4.5 Segmentation software

The present quantitative results demonstrate the clinical validity of all four segmentation methods SCT_PS, SCT_DS, ASM and NQL. All four are suitable for discriminating between rMS and pMS, at the C1-2 and C1-7 vertebral levels, and for spinal cord MRI and brain MRI (for C1-2), with the overall highest AUC for C1-C2 height normalized CSA from NQL measured at C1-7 (0.877) and the lowest AUC for unnormalized CSA from SCT_PS measured at C1-2 (0.685) (Table 5). Strong associations between CSA and EDSS scores were also observed across the board, with CSA measured in the entire cervical cord (C1-7) giving the strongest correlations, especially for SCT_PS and NQL (Spearman’s rho of −0.646 and −0.642, both corrected *p* < 0.001, for nCSA_C1-C2_).

This observation of clinical validity of all four segmentation methods occurred despite systematic CSA differences (Table 2; Figure 3) between most pairs of segmentation methods, which confirmed previous findings (18–22). Specifically, the SCT_PS and SCT_DS methods resulted in smaller CSA than ASM and NQL, while ASM and NQL exhibited close agreement, as previously also found in a smaller group of patients with MS and HC (19) and in a multicenter study on a traveling healthy volunteer (18). This systematic CSA difference between segmentation methods is largely related to differences in the definition of the cord edges and the handling of partial volume effects. For example, cord lesions adjacent to the cord edge may be partly classified as CSF by intensity-based algorithms like NQL, leading to effective underestimation of the CSA, while in active surface modeling methods, constraints on the deformation of the surface typically prevent large shape changes due to local lesions on the cord edge, which may lead to a smaller effect on CSA. The higher prevalence of lateral cord lesions in pMS compared with rMS at the C1-2 vertebral level (49) might explain the lower CSA for NQL compared to ASM in pMS (Table 2), and thereby the higher AUC for NQL in discriminating pMS from rMS (ROC analyses in Table 5 and Supplementary Table S8).

Although the observed CSA differences between methods imply that comparing absolute CSA values from different segmentation methods is not possible and results should not be mixed, our present results show that clinical validity of each method alone is not compromised by these systematic differences, and this is an important result for future studies on cord atrophy in MS.

### 4.6 Limitations

Limitations of our study should be discussed. First, we did not use a strict standardization of the MRI protocol settings and parameter timing between the different MRI centers, but only harmonization with regard to image geometry, resolution and contrast weighting. This probably led to an increase of the variability of CSA between different scanners and limited the detectability of disease related effects. On the other hand, this study design reflected a real-world scenario, which is typical for a multi-center setting. Recently, a fully harmonized examination protocol adapted to different scanner types, including sagittal 3D-T1w imaging and other sequences for quantitative MRI of the spinal cord was introduced (the spine generic protocol, https://spinalcordmri.org/protocols). This generic spinal cord protocol has successfully been implemented in 42 MRI centers worldwide in order to generate a harmonized multi-subject dataset (36, 50). Future multi-centric studies on CSA quantification could adopt this approach to acquisition.

This work exclusively used 3D T1-weighted imaging, but other pulse sequences have also been used to measure CSA, including T2-weighted imaging [e.g., (51)] and phase-sensitive inversion recovery (PSIR) [e.g., (28)]. It would be useful to confirm clinical validity for such other image types to further enhance widespread clinical applicability.

Another limitation is that this work investigated only a selection of measures for normalizing CSA values and did not include any head or brain derived measures.

Further, in our ROC analyses of impact of the different measurement strategies on differentiating between rMR and pMS subtypes we did not perform full testing across all strategies and contrasts. Instead, we mainly focussed on the impact of normalization in single, pairwise comparisons between normalized and unnormalized CSA. To test the effects of other methodologic variables, we analysed a small selection of contrasts (ASM and NQL methods, C1-2 and C1-7 levels, brain or cord MRI and normalization) and partly confirmed the results of the latter ROC analysis regarding normalization and better discrimination using NQL. Future work should include full contrast analyses to assess differentiation between MS subtypes.

Although center related factors, such as scanner type, acquisition protocol, demographics might influence our results, we did not primarily include investigation of center effects in our group comparisons and ROC analyses, because in our study the MS subtypes were distributed unevenly between the centers and inclusion of center as a confounder would have diminished the differences between the subgroups. We acknowledge this as a limitation. Still, we presumed that these center related effects were small compared to the differences that we observe between HC and MS patient groups. We regard this as justified because marked MS related CSA atrophy has been consistently reported in the literature, for example in (1, 3, 4, 13), and on the basis of our previous study (18). Therein the same MRI scanners, local protocols and evaluation methods as in the present study were applied to a traveling volunteer. The results showed center related differences in CSA being <1.5 mm^2^ across all methodologies, thus being much smaller than the disease effect. Still, to make this lack transparent and investigate the impact of center effects, we have included an additional set of analyses including center as a confounding variable for the group comparison (HC, MS and MS subtypes, in Supplementary Table S2). Inclusion of center in the covariate adjusted ROC analyses did not allow differentiation between rMS and pMS (all AUC < 0.5).

The measurement methods compared in this study (i.e., the combination of acquisition type, cord region, and software) have all previously been evaluated in terms of repeatability and between-scanner reproducibility in a traveling volunteer (18), and for head acquisition only also in a cohort of people with MS and healthy volunteers (19). The absence of a direct assessment of scan-rescan repeatability within the present study could also be considered a limitation. However, because we considered the added burden to participants of a repeated scan disproportionately high, we have chosen instead to rely on those previous papers to establish the reliability of the methods used.

Furthermore, we did not investigate the operator dependent variability, which is specific to the semi-automated methods ASM and NQL, as these additional analyses would have gone beyond the scope of our study. The inter-rater variability of the ASM and NQL methods has been investigated in previous studies (21, 45) and was <= 1% in both methods.

We did not specifically investigate the effect of lesions on CSA measurements, because previous work on the same software (19) found that the presence of MS lesions in the cord did not diminish accuracy of the segmentations. Therefore, we used those methods here without again confirming in our data that accuracy was not affected by the presence of lesions. The unbalanced distribution of disease types across centers may have impacted the discrimination between disease types based on CSA. The relatively small number of healthy controls, resulting from the requirement of having both head and neck 3D T1-weighted MRI of good quality, is another limitation. Specifically in the estimation of coefficients of variation of CSA in the HC group this may increase the variability of these results. We have therefore estimated the ranges of CV, as shown in Table 3. The relatively large variability of CSA in the rMS group, possibly arising from the large ranges of age and disease duration, limited our ability to investigate differences between rMS and HC.

Lastly, this study did not investigate longitudinal atrophy rates, which are more important than cross-sectional CSA values in the context of clinical trials or longitudinal patient monitoring. While CSA may be important for cross-sectional patient characterization and group comparisons, reliable measurements of change over time are for example needed for estimations of required sample sizes in a clinical trial in which an active treatment reduces CSA atrophy over time. Although subtraction of two cross-sectional CSA measurements is easy to use for atrophy estimation, this approach tends to accumulate variability: by the inherent inaccuracies of the single cross-sectional estimations, and due to differences between the time-points regarding image quality, patient positioning and cord curvature within the coil, and possible variations in scanner settings. Therefore, when measuring longitudinal SC atrophy progression, recently proposed registration based methods, such as the GBSI method, the longitudinal version of the ASM method (using pair-wise registration), the SCORE method or the SIENA-SC method would be preferable (45, 52–54).

## 5 Conclusion

Whole-cervical cord (C1-7) CSA measurements typically performed better than C1-2 CSA measurements; normalizing by C1-C2 or C1-C3 vertebral height improved performance; and performance varied between image analysis methods depending on the setting. The quantitative results obtained may support future study design decisions for multi-center studies to be made informed by relevant quantitative data. To facilitate this, recommendations were derived and summarized in Figure 5 and Supplementary Table S8.

## Data Availability

The datasets presented in this article are not readily available because the data on which this multicentre study is based are the property of the respective participating institutions. Access to the data can be mediated by the MAGNIMS steering committee upon request. Requests to access the datasets should be directed to https://www.magnims.eu/.
