# Supplementary material for: Optimization of cervical cord atrophy measurement using a real-world, multicentre dataset in multiple sclerosis
Source: Front Neurol. 2025 Nov 26;16:1657484. doi: 10.3389/fneur.2025.1657484 (PMC12689376; doi:10.3389/fneur.2025.1657484)
Supplement: Supplementary file 1 [file Data_Sheet_1.pdf]

frontiers

# *Supplementary Material*

**Title: Optimization of cervical cord atrophy measurement using a real-world multi-center dataset in Multiple Sclerosis**

**Description the software methods for cervical cord segmentation**

Calculation of cord area (CSA) and longitudinal cord area changes: for CSA measurements, four different software methods were used, that have previously been used in large MS cohorts (Rocca et al. 2011; Lukas et al. 2015; Prados et al. 2016, Moccia et al. 2020). The first approach was the active surface model (ASM), a semiautomatic software method commercially available with the Jim Software package (“ cord finder” tool in JIM, v. 7.0 Xinapse Systems, Colchester, UK; www.xinapse.com)(Horsfield, Sala et al. 2010, Rocca, Horsfield et al. 2011), the second semiautomatic method was the watershed-segmentation method available with NeuroQlab (NQL, Fraunhofer-Mevis, Bremen, Germany; license freely available for research purposes upon request from Fraunhofer-Mevis) (Lukas, Sombekke et al. 2013, Weiler 2017). In addition to these semiautomatic approaches, two automatic software methods were chosen: the deformable model method PropSeg, and the DeepSeg tool that is based on convolutional neural networks, both freely available with the Spinal cord Toolbox (SCT) (De Leener, Kadoury et al. 2014, De Leener, Levy et al. 2017, Gros, De Leener et al. 2019) (https://spinalcordtoolbox.com).

<u>ASM (JIM software):</u>

The Active Surface tool requires interactive marking of the center of the cord at a regular distance along several vertebral levels to be included in the analysis (Horsfield, Sala et al. 2010). Cord center-line and cord outlines at each slice are then calculated using a segmentation algorithm with a steadily increasing refinement of the active surface model. This allows a rapid semi-automated segmentation by measuring the cord CSA along the length of the extracted surface. We used the cord finder tool included in JIM version 7.0, with the following settings: nominal cord diameter setting: 10 mm, number of shape coefficients: 18 and order of longitudinal variation: 5. ASM has been shown to be highly reproducible and the method has been used in previous cross sectional and in longitudinal MS studies (Rocca, Horsfield et al. 2011, Valsasina, Rocca et al. 2013, Valsasina, Rocca et al. 2015, Rocca, Valsasina et al. 2019, Weeda, Middelkoop et al. 2019, Bischof, Papinutto et al. 2022, Valsasina, Gobbi et al. 2022).

<u>NQL:</u>

NQL (version 4.01) requires as the first step to define interactively the section of the cord to be analysed by placing an oblique plane through the dataset, which runs through the upper and lower end of the section (Weiler 2017). This is aided by two perpendicular lines, which allow precise alignment of the section to the specific vertebral bodies. This step is followed by a semi-automatic pre-segmentation using a watershed transformation of the pixel intensities. Subsequently, a fully automated model based volume measurement is performed by fitting the intensity distribution of the pre-segmented input region using a Gaussian mixture model. The spinal cord volume is modelled using Gaussian mixture of two tissue classes (spinal cord tissue and CSF) and a separate class

representing partial volume voxels. The volume is calculated by summation of the spinal cord tissue class volume and half of the volume of the partial volume class. The center-line of the SC is calculated and used to determine the mean CSA by normalizing the measured volume to the section length. The operator can correct the final results interactively. NQL has been shown to be highly reproducible (Lukas, Sombekke et al. 2013, Weiler 2017) and the method has already been used in cross-sectional and in longitudinal studies of different neurological diseases including MS (Lukas, Hahn et al. 2008, Daams, Weiler et al. 2014, Lukas, Knol et al. 2015, Liu, Lukas et al. 2016, Schneider, Bellenberg et al. 2017, Wilhelms, Bellenberg et al. 2017, Weeda, Middelkoop et al. 2019).

SCT PropSeg (SCT_PS)

The Spinal Cord Toolbox SCT features specific segmentation tools for the spinal cord. The segmentation algorithm PropSeg is based on an iterative propagation of a deformable model with adaptive contrast mechanism (De Leener, Kadoury et al. 2014, De Leener, Levy et al. 2017). Automated detection of the center of the spinal cord is done by ellipse detection and information from the body symmetry, followed by propagating a tubular surface along the spinal cord edge using deformable models. SCT_PS has been applied in studies on MS patients (Yiannakas, Mustafa et al. 2016) and has been shown to be highly reproducible (McCoy, Dupont et al. 2019).

SCT DeepSeg (SCT_DS)

The DeepSeg algorithm implemented in SCT is based on a deep learning convolutional neural network (CNN) module trained to effectively segment the spinal cord from MRI images. SCT_DS was trained from masks generated by SCT_PS, which were manually corrected if necessary (De Leener, Levy et al. 2017, Gros, De Leener et al. 2019). SCT_DS has been thoroughly evaluated in a large multi-centre dataset of HC and has been applied in different diseases related to cord atrophy (McCoy, Dupont et al. 2019, Moccia, Valsecchi et al. 2020, Bautin and Cohen-Adad 2021).

We used SCT (version 5.6, https://spinalcordtoolbox.com) with default settings to segment the cervical cord and automatically identify different vertebral levels in SCT_PS and SCT_DS. The segmentation and labeling quality was visually inspected using the implemented quality control tool. Problems with automatic vertebral labeling in single cases were resolved by using the dedicated manual labeling procedure.

Average operator times required for a single CSA measurement

For the semiautomated methods ASM and NQL, although the computation times for spinal cord segmentation are fast (< 1 min), average operator times for a single CSA measurement including potential error correction, and estimation of normalization measures are 8-10 minutes.

For the automated methods of Spinal Cord Toolbox SCT_DS and SCT_PS the speed of the spinal cord segmentation and output of results directly depends on the computing power of the system used and for example takes 7 minutes for cord segmentation using a standalone computer equipped with quad-core Intel Core i5 64-bit 1.4 GHz CPU and Memory 8 GB 2133 MHz. After automatic processing of a bulk of examinations additional operator time of 2-3 minutes per scan is required for quality control and estimation of normalization measures.

Consideration of partial volume effects and of cord curvature in the different software methods:

As laid out in detail in our previous methodological study, the methods have specific differences related to the definition of the cord edges and handling of partial volume effects at the transition between cord and surrounding cerebrospinal fluid (CSF)(Lukas, Bellenberg et al. 2021). In addition, the effects of cord curvature are treated differently between ASM and NQL or SCT_PS and SCT_DS. Due to the limited image resolution and the small diameter of the SC these effects may account for a substantial proportion of the segmented volume (Yiannakas, Mustafa et al. 2016, Weeda, Middelkoop et al. 2019, Lukas, Bellenberg et al. 2021).

The SCT_PS algorithm includes only voxels into the segmentation that are classified as "pure" cord tissue, without referring to partial volume effects at the margin of the cord contour. As SCT_DS segmentation was trained on SCT_PS results, the method has the same characteristic as SCT_PS and excludes voxels that are prone to partial volume effects. In contrast, NQL includes pure cord tissue voxels and 50% of the partial-volume tissue class in the CSA calculation (Weiler 2017, Yiannakas, Mustafa et al. 2016). Similarly to NQL, the cord segmentation of ASM includes a fraction of those voxels subject to partial volume effects between the cord and the surrounding CSF because the cord surface definition is partly controlled by seeking high intensity gradients (Horsfield, Sala et al. 2010). Thus, CSA results of ASM and NQL are typically higher than CSA generated by SCT_PS or SCT_DS.

Additionally, effects of the cervical cord curvature are treated differently between NQL and ASM or SCT. While ASM and SCT methods are optimized with regards to variations of the cord curvature (Horsfield, Sala et al. 2010, De Leener, Kadoury et al. 2014), NQL quantifies the cord volume between two parallel oblique planes, and uses the centerline merely for calculation of the mean cord area from the segmented volume (Weiler 2017). Thus, the CSA estimations by NQL might differ from the corresponding ASM or SCT results, depending of the degree of curvature of the SC, or the exact choice of the cord section to be analyzed.

**References (Supplement)**

Bautin, P. & Cohen-Adad, J. 2021. Minimum detectable spinal cord atrophy with automatic segmentation: Investigations using an open-access dataset of healthy participants. Neuroimage Clin, 32, 102849.

Bischof, A., Papinutto, N., Keshavan, A., Rajesh, A., Kirkish, G., Zhang, X., Mallott, J. M., Asteggiano, C., Sacco, S., Gundel, T. J., Zhao, C., Stern, W. A., Caverzasi, E., Zhou, Y., Gomez, R., Ragan, N. R., Santaniello, A., Zhu, A. H., Juwono, J., Bevan, C. J., Bove, R. M., Crabtree, E., Gelfand, J. M., Goodin, D. S., Graves, J. S., Green, A. J., Oksenberg, J. R., Waubant, E., Wilson, M. R., Zamvil, S. S., University of California, S. F. M. S. E. T., Cree, B. a. C., Hauser, S. L. & Henry, R. G. 2022. Spinal Cord Atrophy Predicts Progressive Disease in Relapsing Multiple Sclerosis. Ann Neurol, 91, 268-281.

Daams M, Weiler F, Steenwijk MD, Hahn HK, Geurts JJ, Vrenken H, et al. Mean upper cervical cord area (MUCCA) measurement in long-standing multiple sclerosis: relation to brain findings and clinical disability. Mult Scler J. (2014) 20:1860–5.

De Leener, B., Kadoury, S. & Cohen-Adad, J. 2014. Robust, accurate and fast automatic segmentation of the spinal cord. Neuroimage, 98, 528-36.

De Leener, B., Levy, S., Dupont, S. M., Fonov, V. S., Stikov, N., Louis Collins, D., Callot, V. & Cohen-Adad, J. 2017. SCT: Spinal Cord Toolbox, an open-source software for processing spinal cord MRI data. Neuroimage, 145, 24-43.

Gros, C., De Leener, B., Badji, A., Maranzano, J., Eden, D., Dupont, S. M., Talbott, J., Zhuoquiong, R., Liu, Y., Granberg, T., Ouellette, R., Tachibana, Y., Hori, M., Kamiya, K., Chougar, L., Stawiarz, L., Hillert, J., Bannier, E., Kerbrat, A., Edan, G., Labauge, P., Callot, V., Pelletier, J., Audoin, B., Rasoanandrianina, H., Brisset, J. C., Valsasina, P., Rocca, M. A., Filippi, M., Bakshi, R., Tauhid, S., Prados, F., Yiannakas, M., Kearney, H., Ciccarelli, O., Smith, S., Treaba, C. A., Mainero, C., Lefeuvre, J., Reich, D. S., Nair, G., Auclair, V., Mclaren, D. G., Martin, A. R., Fehlings, M. G., Vahdat, S., Khatibi, A., Doyon, J., Shepherd, T., Charlson, E., Narayanan, S. & Cohen-Adad, J. 2019. Automatic segmentation of the spinal cord and intramedullary multiple sclerosis lesions with convolutional neural networks. Neuroimage, 184, 901-915.

Horsfield, M. A., Sala, S., Neema, M., Absinta, M., Bakshi, A., Sormani, M. P., Rocca, M. A., Bakshi, R. & Filippi, M. 2010. Rapid semi-automatic segmentation of the spinal cord from magnetic resonance images: application in multiple sclerosis. Neuroimage, 50, 446-55.

Liu, Y., Lukas, C., Steenwijk, M. D., Daams, M., Versteeg, A., Duan, Y., Li, K., Weiler, F., Hahn, H. K., Wattjes, M. P., Barkhof, F. & Vrenken, H. 2016. Multicenter Validation of Mean Upper Cervical Cord Area Measurements from Head 3D T1-Weighted MR Imaging in Patients with Multiple Sclerosis. AJNR Am J Neuroradiol, 37, 749-54.

Lukas, C., Knol, D. L., Sombekke, M. H., Bellenberg, B., Hahn, H. K., Popescu, V., Weier, K., Radue, E. W., Gass, A., Kappos, L., Naegelin, Y., Uitdehaag, B. M., Geurts, J. J., Barkhof, F. & Vrenken, H. 2015. Cervical spinal cord volume loss is related to clinical disability progression in multiple sclerosis. J Neurol Neurosurg Psychiatry, 86, 410-8.

Lukas, C., Bellenberg, B., Prados, F., Valsasina, P., Parmar, K., Brouwer, I., Pareto, D., Rovira, A., Sastre-Garriga, J., Gandini Wheeler-Kingshott, C. a. M., Kappos, L., Rocca, M. A., Filippi, M., Yiannakas, M., Barkhof, F. & Vrenken, H. 2021. Quantification of Cervical Cord Cross-Sectional Area: Which Acquisition, Vertebra Level, and Analysis Software? A Multicenter Repeatability Study on a Traveling Healthy Volunteer. Front Neurol, 12, 693333.

Lukas, C., Hahn, H. K., Bellenberg, B., Hellwig, K., Globas, C., Schimrigk, S. K., Koster, O. & Schols, L. 2008. Spinal cord atrophy in spinocerebellar ataxia type 3 and 6 : impact on clinical disability. J Neurol, 255, 1244-9.

Lukas, C., Sombekke, M. H., Bellenberg, B., Hahn, H. K., Popescu, V., Bendfeldt, K., Radue, E. W., Gass, A., Borgwardt, S. J., Kappos, L., Naegelin, Y., Knol, D. L., Polman, C. H., Geurts, J. J., Barkhof, F. & Vrenken, H. 2013. Relevance of spinal cord abnormalities to clinical disability in multiple sclerosis: MR imaging findings in a large cohort of patients. Radiology, 269, 542-52.

McCoy DB, Dupont SM, Gros C, Cohen-Adad J, Huie RJ, Ferguson A, Duong-Fernandez X, Thomas LH, Singh V, Narvid J, Pascual L, Kyritsis N, Beattie MS, Bresnahan JC, Dhall S,

Whetstone W, Talbott JF; TRACK-SCI Investigators. Convolutional Neural Network-Based Automated Segmentation of the Spinal Cord and Contusion Injury: Deep Learning Biomarker Correlates of Motor Impairment in Acute Spinal Cord Injury. AJNR Am J Neuroradiol. 2019 Apr;40(4):737-744.

Moccia, M., Valsecchi, N., Ciccarelli, O., Van Schijndel, R., Barkhof, F. & Prados, F. 2020. Spinal cord atrophy in a primary progressive multiple sclerosis trial: Improved sample size using GBSI. Neuroimage Clin, 28, 102418.

Prados, F., Cardoso, M. J., Yiannakas, M. C., Hoy, L. R., Tebaldi, E., Kearney, H., Liechti, M. D., Miller, D. H., Ciccarelli, O., Wheeler-Kingshott, C. A. & Ourselin, S. 2016. Fully automated grey and white matter spinal cord segmentation. Sci Rep, 6, 36151.

Rocca, M. A., Horsfield, M. A., Sala, S., Copetti, M., Valsasina, P., Mesaros, S., Martinelli, V., Caputo, D., Stosic-Opincal, T., Drulovic, J., Comi, G. & Filippi, M. 2011. A multicenter assessment of cervical cord atrophy among MS clinical phenotypes. Neurology, 76, 2096-102.

Rocca, M. A., Valsasina, P., Meani, A., Gobbi, C., Zecca, C., Rovira, A., Montalban, X., Kearney, H., Ciccarelli, O., Matthews, L., Palace, J., Gallo, A., Bisecco, A., Gass, A., Eisele, P., Lukas, C., Bellenberg, B., Barkhof, F., Vrenken, H., Preziosa, P., Comi, G., Filippi, M. & Group, M. S. 2019. Clinically relevant cranio-caudal patterns of cervical cord atrophy evolution in MS. Neurology, 93, e1852-e1866.

Schneider R, Bellenberg B, Kleiter I, Gold R, Köster O,Weiler F, et al. Cervical cord and ventricle affection in neuromyelitis optica. Acta Neurol Scand. (2017) 135:324–31. doi: 10.1111/ane.12601

Valsasina P, Gobbi C, Zecca C, Rovira A, Sastre-Garriga J, Kearney H, Yiannakas M, Matthews L, Palace J, Gallo A, Bisecco A, Gass A, Eisele P, Filippi M, Rocca MA; MAGNIMS Study Group;. Characterizing 1-year development of cervical cord atrophy across different MS phenotypes: A voxel-wise, multicentre analysis. Mult Scler. 2022 May;28(6):885-899.

Valsasina, P., Rocca, M. A., Horsfield, M. A., Absinta, M., Messina, R., Caputo, D., Comi, G. & Filippi, M. 2013. Regional cervical cord atrophy and disability in multiple sclerosis: a voxel-based analysis. Radiology, 266, 853-61.

Valsasina P, Rocca MA, Horsfield MA, Copetti M, Filippi M. A longitudinal MRI study of cervical cord atrophy in multiple sclerosis. J Neurol. (2015) 262:1622–8. doi: 10.1007/s00415-015-7754-z

Weeda, M. M., Middelkoop, S. M., Steenwijk, M. D., Daams, M., Amiri, H., Brouwer, I., Killestein, J., Uitdehaag, B. M. J., Dekker, I., Lukas, C., Bellenberg, B., Barkhof, F., Pouwels, P. J. W. & Vrenken, H. 2019. Validation of mean upper cervical cord area (MUCCA) measurement techniques in multiple sclerosis (MS): High reproducibility and robustness to lesions, but large software and scanner effects. Neuroimage Clin, 24, 101962.

Weiler, F., Hallmann, M. F., Schwier, M., Hildebrandt, H., Gregori, J., Spiess, L., Klein, J. & Heldmann, S. 2017. Fully automated detection, segmentation and quantification of mean cross-sectional area of the spinal cord. Multiple Sclerosis Journal, 23, 149-150.

Wilhelms W, Bellenberg B, Koster O, Weiler F, Hoffmann R, Gold R, et al. Progressive spinal cord atrophy in manifest and premanifest huntington's disease. J Neurol Neurosurg Psychiatry. (2017) 88:614–6.

Yiannakas, M. C., Mustafa, A. M., De Leener, B., Kearney, H., Tur, C., Altmann, D. R., De Angelis, F., Plantone, D., Ciccarelli, O., Miller, D. H., Cohen-Adad, J. & Gandini Wheeler-Kingshott, C. A. 2016. Fully automated segmentation of the cervical cord from T1-weighted MRI using PropSeg: Application to multiple sclerosis. Neuroimage Clin, 10, 71-7.

## Supplementary Figures and Tables

### Supplementary Figures

**Supplementary Figure S1:** Example of receiver operating characteristic (ROC) curves for the differentiation between rMS and pMS using unnormalized or normalized CSA measured at the C1-7 vertebral level using the method ASM and NQL (upper row), SCT_DS and SCT_PS (lower row); color coding: black: unnormalized CSA, green: C1-C2 normalized CSA ($nCSA_{C1-C2}$), blue: C1-C3 normalized CSA ($nCSA_{C1-C3}$), orange: C1-C7 normalized CSA ($nCSA_{C1-C7}$), green: C1-C2 normalized CSA ($nCSA_{C1-C2}$), purple: C3-area normalized CSA ($nCSA_{C3-area}$)

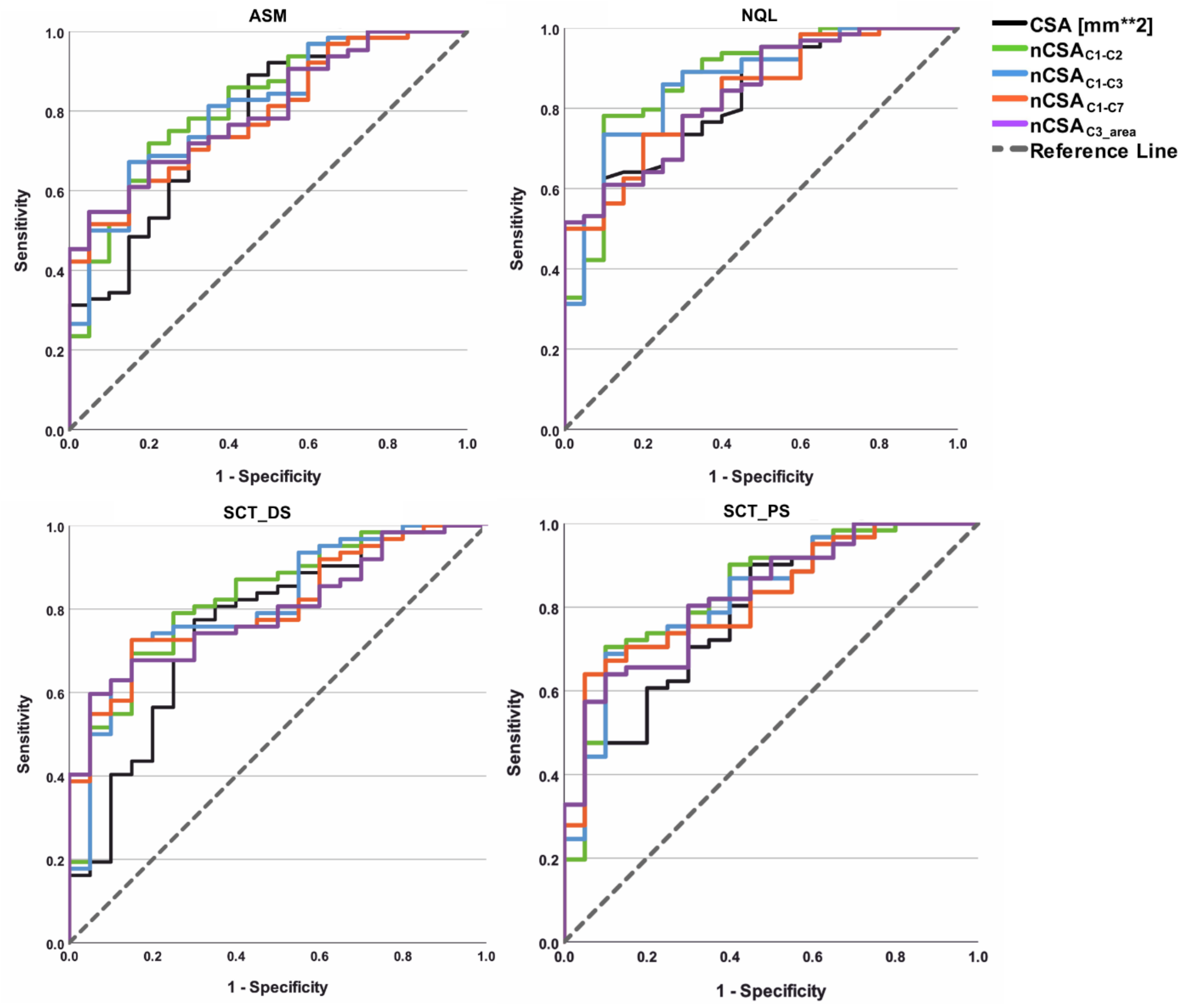

**Supplementary Tables**

**Supplementary Table S1:** Sequence parameters of 3D-T1w imaging used with the 3Tesla scanners at the participating centers

| **Site Nr** | **1** | **2** | **3** | **4** | **5** |
|---|---|---|---|---|---|
| Vendor | GE Discovery MR750 | Siemens Trio Tim | Philips Achieva | Philips Intera | Philips Achieva |
| Magnet length / bore diameter [cm] | 194 /60 | 198 / 60 | 157 / 60 | 157 / 60 | 157 / 60 |
| Coil type | HNS array 16 ch. | head-neck array 16 ch. | SENSE-NV-16 ch. | SENSE-NV-16 ch. | SENSE-NV-16 ch. |
| **Head MRI (3D-T1-weighted)** | | | | | |
| Sequence | FSPGR | MPRAGE | TFE | TFE | TFE |
| Number of Slices | 172 | 192 | 180 | 204 | 180 |
| Orientation | sagittal | sagittal | Sagittal | Sagittal | sagittal |
| TR (ms) | 7.8 | 2300 | 10 | 7.1 | 6.8 |
| TE (ms) | 3 | 3 | 4.6 | 3.2 | 3.1 |
| TI (ms) | 450 | 900 | 1000 | 900 | 825 |
| FA | 12° | 9° | 8° | 9° | 8° |
| Voxel Size (mm) | 0.98×0.98×1 | 1×1×1 | 1x1x1 | 1x1x1 | 1x1x1 |
| **Cervical cord MRI (3D-T1-weighted)** | | | | | |
| Sequence | FSPGR | MPRAGE | TFE | TFE | TFE |
| Number of Slices | 172 | 128 | 64 | 64 | 128 |
| Orientation | sagittal | sagittal | sagittal | sagittal | sagittal |
| TR (ms) | 7.3 | 2300 | 8 | 8 | 8 |
| TE (ms) | 3 | 3.26 | 3.5 | 3.5 | 3.7 |
| TI (ms) | 450 | 900 | 1000 | 1000 | 856 |
| FA | 15° | 9° | 8° | 8° | 8° |
| Voxel Size (mm) | 1×1×1 | 1×1×1 | 1x1x1 | 1x1x1 | 1x1x1 |

**Abbreviations:** TR Repetition time; TE Echo time. TI Inversion time; FA Flip angle; TFE Turbo field echo; MPRAGE Magnetization prepared rapid acquisition gradient echo; FSPGR Fast spoiled gradient recalled echo; Site Numbers: 1= Amsterdam UMC, Amsterdam; 2= Hospital Universitari Vall d’Hebron Barcelona; 3= Ruhr-University of Bochum; Bochum; 4= IRCCS San Raffaele Scientific Institute; Milano; 5= University College London; London.

**Supplementary Table S2:** Cord cross-sectional area (CSA unnormalized) in MS subgroups and HC for different software, cervical cord levels and brain or cord MRI. P-value and effect size calculation between groups was adjusted for center, age and sex.

| | **method** | **ASM** | | | **NQL** | | | **SCT_PS** | | | **SCT_DS** | | |
|---|---|---|---|---|---|---|---|---|---|---|---|---|---|
| | **vertebral level** | **C1-2** | **C1-2** | **C1-7** | **C1-2** | **C1-2** | **C1-7** | **C1-2** | | | **C1-2** | **C1-2** | **C1-7** |
| **CSA [mm²] mean ± SD** | MRI type | brain | cervical cord | cervical cord | brain | cervical cord | cervical cord | brain | cervical cord | cervical cord | brain | cervical cord | cervical cord |
| | HC | 82.4 ± 9.6 | 83.9 ± 11.1 | 79.1± 9.9 | 82.7 ± 9.6 | 82.4 ± 9.6 | 82.7 ± 9.5 | 72.4 ± 7.0 | 74.0 ± 7.8 | 74.8 ± 9.0 | 68.2 ± 7.6 | 64.5 ± 8.0 | 62.9± 8.5 |
| | MS | 75.4 ± 9.1 | 76.6 ± 9.7 | 72.6 ± 9.7 | 75.0 ± 9.3 | 76.0 ± 10.0 | 76.0 ± 10.7 | 66.3 ± 8.9 | 68.5 ± 10.3 | 69.6 ± 11.9 | 61.5 ± 8.8 | 60.5 ± 9.1 | 58.8 ± 8.2 |
| | rMS | 77.0 ± 8.5 | 78.4 ± 9.1 | 74.9 ± 8.5 | 77.1 ± 8.7 | 78.4 ± 8.9 | 79.1 ± 8.6 | 67.9 ± 8.5 | 70.5 ± 9.7 | 72.7± 10.7 | 63.4± 8.3 | 62.3 ± 8.7 | 60.7 ± 7.3 |
| | pMS | 70.2 ± 8.9 | 70.9 ± 9.6 | 65.3 ± 9.6 | 68.2 ± 8.0 | 68.0 ± 9.3 | 66.0 ± 10.6 | 61.3 ± 8.6 | 62.4 ± 9.8 | 59.9 ± 10.8 | 55.8 ± 8.0 | 54.9 ± 8.1 | 52.8 ± 8.1 |
| **HC - MS** | p (HC-MS) | 0.12 | 0.12 | 0.12 | 0.12 | 0.197 | 0.275 | 0.12 | 0.236 | 0.549 | 0.12 | 0.185 | 0.129 |
| | Effect size η2 (95% CI) | 0.07 (0.00;0.25) | 0.08 (0.00;0.25) | 0.07 (0.00;0.24) | 0.09 (0.00;0.26) | 0.04 (0.00;0.19) | 0.03 (0.00;0.17) | 0.07 (0.00;1.0) | 0.03 (0.00;0.92) | 0.01 (0.00;0.12) | 0.10 (0.00;0.27) | 0.05 (0.00;0.21) | 0.06 (0.00;0.23) |
| **HC – rMS - pMS** | p (HC-pMS)[a] | **0.024** | 0.08 | **0.034** | **0.012** | **0.021** | **0.012** | 0.062 | 0.366 | 0.344 | **0.021** | 0.096 | 0.096 |
| | Effect size η2 (95% CI) | 0.32 (0.06;0.55) | 0.28 (0.01;0.56) | 0.34 (0.05;0.59) | 0.41 (0.15;0.61) | 0.37 (0.09;0.60) | 0.44 (0.17;0.64) | 0.29 (0.02;0.55) | 0.15 0.01;0.48) | 0.18 (0.01;0.53) | 0.37 (0.10;0.59) | 0.22 (0.0;0.53) | 0.29 (0.00;0.59) |
| | p (HC-rMS)[a] | 0.566 | 0.566 | 0.566 | 0.566 | 0.888 | 0.999 | 0.556 | 0.888 | 0.999 | 0.556 | 0.888 | 0.739 |
| | Effect size η2 (95% CI) | 0.07 (0.00;0.25) | 0.07 (0.00;0.23) | 0.05 (0.00;0.21) | 0.07 (0.00;0.24) | 0.03 (0.01;0.17) | 0.02 (0.0;0.15) | 0.06 (0.00;0.23) | 0.03 (0.00;0.17) | 0.04 (0.00;0.11) | 0.10 (0.00;0.27) | 0.03 (0.00;0.18) | 0.04 (0.00;0.20) |
| | p (rMS-pMS)[a] | 0.196 | 0.458 | 0.196 | 0.084 | 0.084 | **0.048** | 0.405 | 0.917 | 0.458 | 0.196 | 0.458 | 0.458 |
| | Effect size η2 (95% CI) | 0.15 (0.00;0.25) | 0.11 (0.00;0.40) | 0.19 (0.00;0.46) | 0.24 (0.03;0.46) | 0.27 (0.04;0.50) | 0.36 (0.12;0.56) | 0.12 (0.00;0.39) | 0.06 (0.00;0.37) | 0.14 (0.00;0.48) | 0.16 (0.00;0.46) | 0.11 (0.00;0.42) | 0.15 (0.00;0.47) |

**Abbreviations**: HC healthy controls, rMS relapsing MS, pMS progressive MS, SCT_DS SCT_deepseg, SCT_PS SCT_propseg, NQL NeuroQLab, ASM active surface method. **p:** all p-values extracted by linear mixed effect models adjusting for age and sex with center as

random intercept; **a** : pairwise contrast with Bonferroni correction for MS subgroup analyses; all p-values corrected for multiple comparisons between methodologies (Benjamini-Holm correction across methods, MRI type, vertebral level); $p$-values $< 0.05$ are marked in bold font; Effect size $\eta 2$: partial eta squared of CSA differences between groups.

**Supplementary Table S3:** Linear regression (general linear model) with CSA or EDSS as dependent variables with inclusion of age as covariate, sex (male=0, female=1) as fixed factor and scanner (center no. 1,2,3,4,5) as a random factor.

| | **HC** | | | **MS** | | | **MS** | | |
|---|---|---|---|---|---|---|---|---|---|
| | **CSA** | | | **CSA** | | | **EDSS** | | |
| Parameter | Regression Coefficient B | Std.-Error | p | Regression Coefficient B | Std.-Error | p | Regression Coefficient B | Std.-Error | p |
| | | | | | | | | | |
| constant | 79.18 | 10.701 | <0.001 | 61.78 | 6.827 | <0.001 | 3.29 | 1.015 | 0.002 |
| age [years] | -0.032 | 0.323 | 0.923 | -0.066 | 0.103 | 0.525 | 0.05 | 0.015 | 0.003 |
| sex | -1.64 | 8.386 | 0.848 | 15.57 | 4.669 | 0.001 | 0.36 | 0.696 | 0.61 |
| [center=1] | -0.74 | 10.981 | 0.947 | 14.94 | 4.675 | 0.002 | -2.42 | 0.697 | 0.001 |
| [center=2] | n.a. | n.a. | n.a. | 19.04 | 5.057 | <0.001 | -3.36 | 0.754 | <0.001 |
| [center=3] | 15.56 | 9.14 | 0.114 | 17.51 | 5.477 | 0.002 | -3.02 | 0.801 | <0.001 |
| [center=4] | 0 | n.a. | n.a. | 16.44 | 4.685 | 0.001 | -1.75 | 0.699 | 0.014 |
| [center=5] | n.a. | n.a. | n.a. | 0 | n.a. | n.a. | 0 | n.a. | n.a. |

Abbreviations: HC: healthy controls, MS: multiple sclerosis, Std.: standard, p: significance, n.a.: not applicable.

**Supplementary Table S4:** Comparison between unnormalized cord cross-sectional area (CSA) using different software methods, separately for brain or cord MRI at different vertebral levels (repeated measure ANOVA with 4 software methods as within-subject factor and Bonferroni correction for multiple comparisons)

| **MRI type** | **Brain MRI** | | **Cord MRI** | | **Cord MRI** | |
|---|---|---|---|---|---|---|
| **vertebral level** | **C1-2** | | **C1-2** | | **C1-7** | |
| Pairwise comparison | Mean CSA difference [95% CI] | Sig. | Mean CSA difference [95% CI] | Sig. | Mean CSA difference [95% CI] | Sig. |
| **SCT_DS vs. SCT_PS** | -4.80 [-5.91;-3.70] | <0.001 | -8.69 [-10.65-6.72] | <0.001 | -11.59 [-14.17;-9.01] | <0.001 |
| **SCT_DS vs. NQL** | -13.51 [-14.42;-12.60] | <0.001 | -15.77 [-17.03;-14.52] | <0.001 | -17.88 [-19.46;-16.30] | <0.001 |
| **SCT_DS vs. ASM** | -14.31 [-15.17;-13.46] | <0.001 | -16.70 [-17.82;-15.57] | <0.001 | -14.48 [-15.65;-13.30] | <0.001 |
| **SCT_PS vs. NQL** | -8.71 [-10.05;-7.36] | <0.001 | -7.09 [-8.87;-5.31] | <0.001 | -6.29 [-8.51;-4.07] | <0.001 |
| **SCT_PS vs. ASM** | -9.51 [-10.79;-8,24] | <0.001 | -8.01 [-9.90;-6.11] | <0.001 | -2.89 [-5.02;-0.75] | 0.003 |
| **NQL vs. ASM** | -0.81 [-1.64;0.03] | 0.065 | -0.92 [-1.81;-0.03] | 0.038 | -3.40 [-4.64;-2.17] | <0.001 |

**Abbreviations:** SCT_DS SCT_deepseg, SCT_PS SCT_propseg, NQL NeuroQLab, ASM active surface method, vs: versus. Sig: p-values of pairwise comparisons using repeated measure ANOVA with 4 software methods as within-subject factor and Bonferroni correction for multiple comparisons; CI: confidence interval.

**Supplementary Table S5:** Comparison of cord cross-sectional area (CSA, unnormalized), **between brain and cord MRI** at the C1-2 vertebral level in MS and HC for different software methods.

| **Method / Vertebral Level** | **MRI type** | **CSA [mm²]** mean ± standard deviation | |
|---|---|---|---|
| | | **MS** | **HC** |
| **ASM** | | | |
| C1-2 | Brain vs. Cord | 75.4 ± 9.1 vs. 76.6 ± 9.7 | 82.4 ± 9.6 vs. 83.9 ± 11.1 |
| Paired t-test | p | <0.001 | 0.076 |
| Intra-class correlation | ICC [95% CI] | 0.979 [0.951;0.989] | 0.992 [0.973;0.997] |
| **NQL** | | **MS** | **HC** |
| C1-2 | Brain vs. Cord | 75.0 ± 9.3 vs. 76.0 ± 10.0 | 82.7 ± 9.6 vs. 82.4 ± 9.6 |
| Paired t-test | p | <0.001 | 0.063 |
| Intra-class correlation | ICC [95% CI] | 0.980 [0.964;0.989] | 0.994 [0.981;0.998] |
| **SCT_PS** | | **MS** | **HC** |
| C1-2 | Brain vs. Cord | 66.3 ± 8.9 vs. 68.5 ± 10.3 | 72.4 ± 7.0 vs. 74.0 ± 7.8 |
| Paired t-test | p | <0.001 | 0.232 |
| Intra-class correlation | ICC [95% CI] | 0.860 [0.728;0.922] | 0.543 [-2.04;0.832] |
| **SCT_DS** | | **MS** | **HC** |
| C1-2 | Brain vs. Cord | 61.5 ± 8.8 vs. 60.5 ± 9.1 | 68.2 ± 7.6 vs. 64.5 ± 8.0 |
| Paired t-test | p | 0.023 | 0.007 |
| Intra-class correlation | ICC [95% CI] | 0.957 [0.931;0.973] | 0.947 [0.760;0.984] |

**Abbreviations:** HC healthy controls, MS Multiple Sclerosis, pMS progressive MS, SCT_DS SCT_deepseg, SCT_PS SCT_propseg, NQL NeuroQLab, ASM active surface method. **p:** p-values: paired t-tests; ICC: intra-class correlation coefficients (two-way mixed effects model for absolute agreement and 95% confidence interval CI)

**Supplementary Table S6: Normalization measures**

Four different normalization measures were derived from a mid-sagittal view of the 3D-T1w cord MRI for each participant at each visit: (1) the height of the C1/C2 vertebra, measured from the tip of C1 to the lower border of the C2 vertebral body in the middle of its anterior-posterior extension; (2) the height of the C1-C3 vertebrae, measured as (1) but down to the lower border of C3 instead of C2; (3) the entire cervical cord length (C1-C7), extracted from the output of the ASM method; and (4) the area of the C3 vertebra. The vertebral heights and dimensions were measured using the ITK-SNAP software viewer (available at www.itksnap.org). Table S4 shows mean (standard deviation) values of the normalization measures within each group.

Table S4: Normalization measures within each group shown as mean (standard deviation)

| Vertebral measure | HC | rMS | pMS | p-value |
|---|---|---|---|---|
| height C1-C2 [mm] | 35.1 (3.0 ) | 33.7 (2.9 ) | 35.4 (2.3 ) | 0.274 |
| height C1-C3 [mm] | 53.0 (4.2 ) | 50.6 (4.4 ) | 52.9 (3.8 ) | 0.172 |
| height C1-C7 [mm] | 125.7 (9.2 ) | 119.3 (10.8 ) | 122.2 (9.2 ) | 0.043 |
| area C3 [mm²] | 228.6 (33.8 ) | 202.0 (40.6 ) | 227.1 (49.8 ) | 0.035 |

Abbreviations: HC healthy controls, rMS relapsing MS, pMS progressive MS. p-values: between-group differences extracted by using analyses of variance (ANCOVA) adjusting for age and sex, using Bonferroni correction for multiple comparisons between subgroups.

In the ANCOVA analyses the differences in the normalization measures between groups were not significant for the height of C1-C2 and height of C1-C3, but significant for height of C1-C7 and the area of C3. There were no between-subjects effects of age, while effects of sex had a significant impact on differences between the groups. This was in line by lower values of the normalization measures in the rMS group, which had a higher fraction of female participants.

**Supplementary Table S7:** Pearson's correlation analyses between unnormalized CSA and the normalization measures in the HC group

| CSA method | Pearson's correlation | height C1-C2 [mm] | height C1-C3 [mm] | height C1-C7 [mm] | area C3 [mm²] |
|---|---|---|---|---|---|
| ASM | r<br>[95% CI] | 0.490<br>[0.187;0.708] | 0.395<br>[0.071;0.643] | 0.284<br>[-0.054;0.584] | -0.217<br>[-0.513;0.125] |
| | p | 0.003 | 0.019 | 0.098 | 0.210 |
| NQL | r<br>[95% CI] | 0.626<br>[0.375;0.792] | 0.557<br>[0.280;0.749] | 0.450<br>[0.142;0.678] | -0.090<br>[-0.407;0.246] |
| | p | <0.001 | <0.001 | 0.006 | 0.602 |
| SCT_PS | r<br>[95% CI] | 0.435<br>[0.129;0.665] | 0.462<br>[0.162;0.683] | 0.436<br>[0.130;0.666] | 0.009<br>[-0.316;0.332] |
| | p | 0.007 | 0.004 | 0.007 | 0.957 |
| SCT_DS | r<br>[95% CI] | 0.541<br>[0.253;0.741] | 0.456<br>[0.145;0.685] | 0.352<br>[0.021;0.613] | -0.089<br>[0.410;0.251] |
| | p | <0.001 | <0.001 | 0.038 | 0.610 |

**Abbreviations:** SCT_DS SCT_deepseg, SCT_PS SCT_propseg, NQL NeuroQLab, ASM active surface method. p: p-values and r: coefficients of Pearson correlations; CI: confidence interval.

**Supplementary Table S8:** Receiver operating characteristic (ROC) analyses of differentiating between rMS and pMS subtypes regarding contrasts between normalized and unnormalized CSA x C1-2 and C1-7 levels x brain and cord MRI x NQL and ASM methods. We show the area under the curve (AUC), 95% confidence interval (CI) and corrected p-values of pair-wise differences between AUC. All p-values derived from pairwise AUC comparisons using DeLong's testing were corrected for multiple comparisons across all contrasts using Benjamini-Hochberg correction.

| | **Method** | **NQL** | | | **ASM** | | |
|---|---|---|---|---|---|---|---|
| | **Vertebral Level** | C1-2 | C1-2 | C1-7 | C1-2 | C1-2 | C1-7 |
| | **MRI type** | brain | cervical cord | cervical cord | brain | cervical cord | cervical cord |
| **CSA** | **AUC CI (95%)** | **0.771 [0.655,0.888]** | **0.791 [0.681,0.901]** | **0.832 [0.737,0.926]** | **0.692 [0.559,0.826]** | **0.716 [0.586,0.846]** | **0.770 [0.648,0.891]** |
| | **p CSA/CSAnorm** | - | - | - | - | - | - |
| | **p C1-2 / C1-7** | - | 0.289 | - | - | 0.179 | - |
| | **p brain / cord** | 0.290 | - | - | 0.370 | - | - |
| | **p NQL / ASM** | 0.050 | 0.046* | 0.064 | - | - | - |
| **nCSA C1-C2** | **AUC CI (95%)** | **0.840 [0.738,0.942]** | **0.852 [0.751,0.952]** | **0.877 [0.786,0.968]** | **0.769 [0.645,0.893]** | **0.777 [0.651,0.902]** | **0.814 [0.706,0.922]** |
| | **p CSA/CSAnorm** | 0.367 | 0.370 | 0.455 | 0.367 | 0.370 | 0.455 |
| | **p C1-2 / C1-7** | - | 0.370 | - | - | 0.370 | - |
| | **p brain / cord** | 0.431 | - | - | 0.691 | - | - |
| | **p NQL / ASM** | 0.070 | 0.064 | 0.064 | - | - | - |
| **nCSA C1-C3** | **AUC CI (95%)** | **0.837 [0.737,0.936]** | **0.843 [0.744,0.942]** | **0.866 [0.775,0.956]** | **0.770 [0.648,0.893]** | **0.769 [0.645,0.892]** | **0.807 [0.701,0.913]** |
| | **p CSA/CSAnorm** | 0.367 | 0.431 | 0.570 | 0.367 | 0.431 | 0.564 |
| | **p C1-2 / C1-7** | - | 0.370 | - | - | 0.370 | - |
| | **p brain / cord** | 0.616 | - | - | 0.961 | - | - |
| | **p NQL / ASM** | 0.138 | 0.138 | 0.064 | - | - | - |
| | **AUC** | **0.812** | **0.829** | **0.834** | **0.739** | **0.752** | **0.788** |

| | | | | | | | |
|---|---|---|---|---|---|---|---|
| | **CI (95%)** | **[0.716,0.908]** | **[0.738,0.920]** | **[0.741,0.926]** | **[0.620,0.858]** | **[0.633,0.872]** | **[0.685,0.892]** |
| **nCSA$_{C1-C7}$** | **p $_{CSA/CSAnorm}$** | 0.540 | 0.564 | 0.961 | 0.540 | 0.594 | 0.748 |
| | **p $_{C1-2 / C1-7}$** | - | 0.875 | - | - | 0.370 | - |
| | **p $_{brain / cord}$** | 0.397 | - | - | 0.651 | - | - |
| | **p $_{NQL / ASM}$** | 0.082 | 0.077 | 0.179 | - | - | - |
| **nCSA$_{C3-area}$** | **AUC CI (95%)** | **0.812 [0.716,0.907]** | **0.825 [0.732,0.918]** | **0.834 [0.741,0.927]** | **0.774 [0.664,0.884]** | **0.774 [0.666,0.882]** | **0.799 [0.700,0.899]** |
| | **p $_{CSA/CSAnorm}$** | 0.665 | 0.701 | 0.961 | 0.456 | 0.570 | 0.729 |
| | **p $_{C1-2 / C1-7}$** | - | 0.616 | - | - | 0.370 | - |
| | **p $_{brain / cord}$** | 0.386 | - | - | 0.961 | - | - |
| | **p $_{NQL / ASM}$** | 0.299 | 0.290 | 0.179 | - | - | - |

**Abbreviations:** SCT_DS: SCT_deepseg, SCT_PS: SCT_propseg, NQL: NeuroQLab, ASM: active surface method, AUC area under the curve p: p-values; CI: confidence interval, CSA, nCSA$_{C1-C2}$, nCSA$_{C1-C3}$, nCSA$_{C1-C7}$, and nCSA$_{C3-area}$ : unnormalized and normalized cervical cross-sectional area

**Supplementary Table S9:** Recommendations for CSA quantification in the cervical cord of patients with MS

<table>
<tr><th>CSA quantification in the cervical spinal cord</th><th colspan="2">Recommendation</th><th colspan="2">Alternative</th></tr>
<tr><td>MRI acquisition type<br><br>Pros and Cons</td><td colspan="2">Dedicated cervical cord MRI with isotropic 3D-T1w<br><br>Pro: *enable measurements at all cervical cord levels;<br>Con: * long acquisition times for brain & cord MRI</td><td colspan="2">Brain MRI incl. sagittal 3D_T1w covering the upper cervical cord<br><br>Pro: * shorter acquisition; better patient comfort;<br>* no significant differences between CSA at C1-2 by brain MRI compared with cord MRI Con: * limited to upper cervical cord sections</td></tr>
<tr><td>CSA in whole cervical cord or upper cervical cord?<br><br>Pros and Cons</td><td colspan="2">Whole cervical cord (C1-7) with cord MRI<br><br>Pro: * Best discrimination between rMS and pMS subtypes; strongest correlations with EDSS scores.<br>Con: * Longer acquisition times (cord MRI needed)</td><td colspan="2">Upper cervical cord sections (e.g. C1-2) with brain or cord MRI<br><br>Pro: *Good discrimination between MS and HC at C1-2 level ;<br>Con: * Slightly worse discrimination between rMS and pMS than whole cervical cord measurements;</td></tr>
<tr><td>CSA normalization<br><br>Pros and Cons</td><td colspan="2">Use spine skeletal normalization metrics: height of C1-C2 vertebrae recommended; optional: height of C1-C3 vertebrae<br><br>Pro: *Using the vertebral height of C1-C2 for CSA normalization improves discrimination between MS subtypes and correlations with EDSS;<br>*easily available as spine MRI derived number;<br>Con: *manual intervention needed.</td><td colspan="2">According to published literature (not part of our study): brain intracranial cavity volume might be used for normalization, if brain MRI and automated analysis pipelines are available.<br>Potential Pros and Cons:<br>Pro: * automated calculation in brain analysis pipelines;<br>Con: * volumetric brain analysis and brain MRI necessary</td></tr>
<tr><td>Image analysis software<br>Suitability dependent on sample size</td><td>Active surface (ASM)<br>Semiautomated</td><td>NQL<br>Semiautomated</td><td>SCT_PS<br>Fully automated</td><td>SCT_DS<br>Fully automated</td></tr>
</table>
